# Genome-wide Association Study Identifies Shared Risk Loci Common to Two Malignancies in Golden Retrievers

**DOI:** 10.1371/journal.pgen.1004922

**Published:** 2015-02-02

**Authors:** Noriko Tonomura, Ingegerd Elvers, Rachael Thomas, Kate Megquier, Jason Turner-Maier, Cedric Howald, Aaron L. Sarver, Ross Swofford, Aric M. Frantz, Daisuke Ito, Evan Mauceli, Maja Arendt, Hyun Ji Noh, Michele Koltookian, Tara Biagi, Sarah Fryc, Christina Williams, Anne C. Avery, Jong-Hyuk Kim, Lisa Barber, Kristine Burgess, Eric S. Lander, Elinor K. Karlsson, Chieko Azuma, Jaime F. Modiano, Matthew Breen, Kerstin Lindblad-Toh

**Affiliations:** 1 Broad Institute of MIT and Harvard, Cambridge, Massachusetts, United States of America; 2 Department of Clinical Sciences, Cummings School of Veterinary Medicine at Tufts University, North Grafton, Massachusetts, United States of America; 3 Science for Life Laboratory, Dept. of Medical Biochemistry and Microbiology, Uppsala University, Uppsala, Sweden; 4 Department of Molecular Biomedical Sciences, College of Veterinary Medicine, & Center for Comparative Medicine and Translational Research, North Carolina State University, Raleigh, North Carolina, United States of America; 5 Department of Veterinary Clinical Sciences, College of Veterinary Medicine, University of Minnesota, Saint Paul, Minnesota, United States of America; 6 Masonic Cancer Center, University of Minnesota, Minneapolis, Minnesota, United States of America; 7 Department of Laboratory Medicine, Boston Children’s Hospital, Boston, Massachusetts, United States of America; 8 Department of Microbiology, Immunology, and Pathology, Colorado State University College of Veterinary Medicine and Biomedical Sciences, Fort Collins, Colorado, United States of America; 9 Animal Cancer Center, Colorado State University College of Veterinary Medicine and Biomedical Sciences, Fort Collins, Colorado, United States of America; 10 FAS Center for Systems Biology, Harvard University, Cambridge, Massachusetts, United States of America; 11 Cancer Genetics Program, University of North Carolina Lineberger Comprehensive Cancer Center, Raleigh, North Carolina, United States of America; University of Bern, SWITZERLAND

## Abstract

Dogs, with their breed-determined limited genetic background, are great models of human disease including cancer. Canine B-cell lymphoma and hemangiosarcoma are both malignancies of the hematologic system that are clinically and histologically similar to human B-cell non-Hodgkin lymphoma and angiosarcoma, respectively. Golden retrievers in the US show significantly elevated lifetime risk for both B-cell lymphoma (6%) and hemangiosarcoma (20%). We conducted genome-wide association studies for hemangiosarcoma and B-cell lymphoma, identifying two shared predisposing loci. The two associated loci are located on chromosome 5, and together contribute ~20% of the risk of developing these cancers. Genome-wide p-values for the top SNP of each locus are 4.6×10^-7^ and 2.7×10^-6^, respectively. Whole genome resequencing of nine cases and controls followed by genotyping and detailed analysis identified three shared and one B-cell lymphoma specific risk haplotypes within the two loci, but no coding changes were associated with the risk haplotypes. Gene expression analysis of B-cell lymphoma tumors revealed that carrying the risk haplotypes at the first locus is associated with down-regulation of several nearby genes including the proximal gene *TRPC6*, a transient receptor Ca^2+^-channel involved in T-cell activation, among other functions. The shared risk haplotype in the second locus overlaps the vesicle transport and release gene *STX8*. Carrying the shared risk haplotype is associated with gene expression changes of 100 genes enriched for pathways involved in immune cell activation. Thus, the predisposing germ-line mutations in B-cell lymphoma and hemangiosarcoma appear to be regulatory, and affect pathways involved in T-cell mediated immune response in the tumor. This suggests that the interaction between the immune system and malignant cells plays a common role in the tumorigenesis of these relatively different cancers.

## Author Summary

To shed light on the genetic predisposition to cancers of the hematologic system, we performed genome-wide association analysis of affected and non-affected pet dogs. Dogs naturally develop the same diseases as humans, including cancer, and the relatively limited genetic diversity within different breeds makes genetic studies easier compared to in humans. By doing genome-wide association, we identified loci predisposing to hemangiosarcoma and B-cell lymphoma. To our surprise, we found two shared loci predisposing to both diseases. Within these two regions we identified several partially overlapping haplotypes, predisposing somewhat differently to the two cancers. We found no coding mutations that followed the risk or non-risk haplotypes suggesting that regulatory mutations exert the effect on disease. We also looked at gene expression in B-cell lymphomas, comparing samples from individuals with risk or non-risk haplotypes. This analysis showed differential expression associated with the haplotypes at both loci, suggesting the risk haplotypes are associated with an effect on T-cell response.

## Introduction

Lymphoma and angiosarcoma are both malignancies of the hematological system, originating from lymphocytes and hematopoietic stem cells, respectively. Lymphomas are a heterogeneous group of diseases, estimated to be the eighth leading cause of human cancer deaths in the US in 2014 [1]. The majority is classified as non-Hodgkin lymphoma (NHL) and, among these, diffuse large B-cell lymphoma (DLBCL) and follicular lymphoma are the most common [2]. Angiosarcoma is a highly aggressive cancer accounting for 1–5% of adult spontaneous sarcomas [3, 4] but its rarity limits genetic studies.

Equivalents of both lymphoma and angiosarcoma occur spontaneously in pet dogs. Sixty-eight percent of golden retrievers, one of the most popular dog breeds in the US, die from cancer [5]. Approximately 13% of golden retrievers develop lymphoma [5], and approximately 50% of these cases are of B-cell origin, within which the most common subtype is the canine equivalent of DLBCL [6–9]. Twenty percent of golden retrievers develop hemangiosarcoma [5], which is clinically and histologically similar to human visceral angiosarcoma [10, 11].

Large-scale population-based epidemiological studies and several genome-wide association studies (GWAS) of human lymphoma cases have shown increased familial risks and germ-line risk factors in the human population [12–15]. These studies provide clear evidence for heritable predisposing mutations for B-cell NHL subtypes in certain human populations, but also point to the heterogeneous nature of B-cell NHL. In this study, we have used the relatively limited genetic diversity in golden retrievers to facilitate the identification of susceptibility loci.

Dogs have been used successfully to map complex diseases including systemic lupus erythematosus, obsessive-compulsive disorder and osteosarcoma [16–19]. Dogs spontaneously develop diseases that are also common in humans, and, as dogs receive modern health care, have recorded family structures and share the living environment with humans, they make an excellent model to study these diseases [8]. In addition, due to recent breed creation, purebred dogs have megabase-sized haplotypes and linkage-disequilibrium (LD) blocks, allowing GWAS in dogs to be performed with 10-fold fewer SNPs than in humans [20, 21]. Power calculations and proof of principle studies have shown that 100–300 cases and 100–300 controls can suffice to map risk factors contributing a 2–5 fold increased risk in dogs [16, 20]. Strong bottlenecks in the evolutionary history of the dog have led to genetic homogeneity within breeds, allowing for relatively efficient identification of germ-line mutations, and allowing for effective clinical trials to study the effect of those germ-line mutations on outcome or response to therapy [22].

Here we present the combined results of GWAS of B-cell lymphoma and hemangiosarcoma in 356 golden retrievers. While originally performed as two separate studies, the major associated regions colocalized, which prompted us to combine the datasets. Our analysis revealed two major loci on canine chromosome 5, associated with both diseases and together accounting for ~20% of the disease risk in this cohort. Neither associated region is explained by coding mutations, but RNA-Seq analysis of differential gene expression in B-cell lymphomas suggests that the risk alleles at the two loci significantly alter expression of genes involved in the T-cell mediated immune response. These results highlight the importance of regulatory mutations, as well as the interaction between the immune system and malignant cells in cancer development, and may explain why these two different diseases unexpectedly share the same predisposing germ-line risk factor.

## Results

### GWAS in hemangiosarcoma and B-cell lymphoma

**Figure 1:**
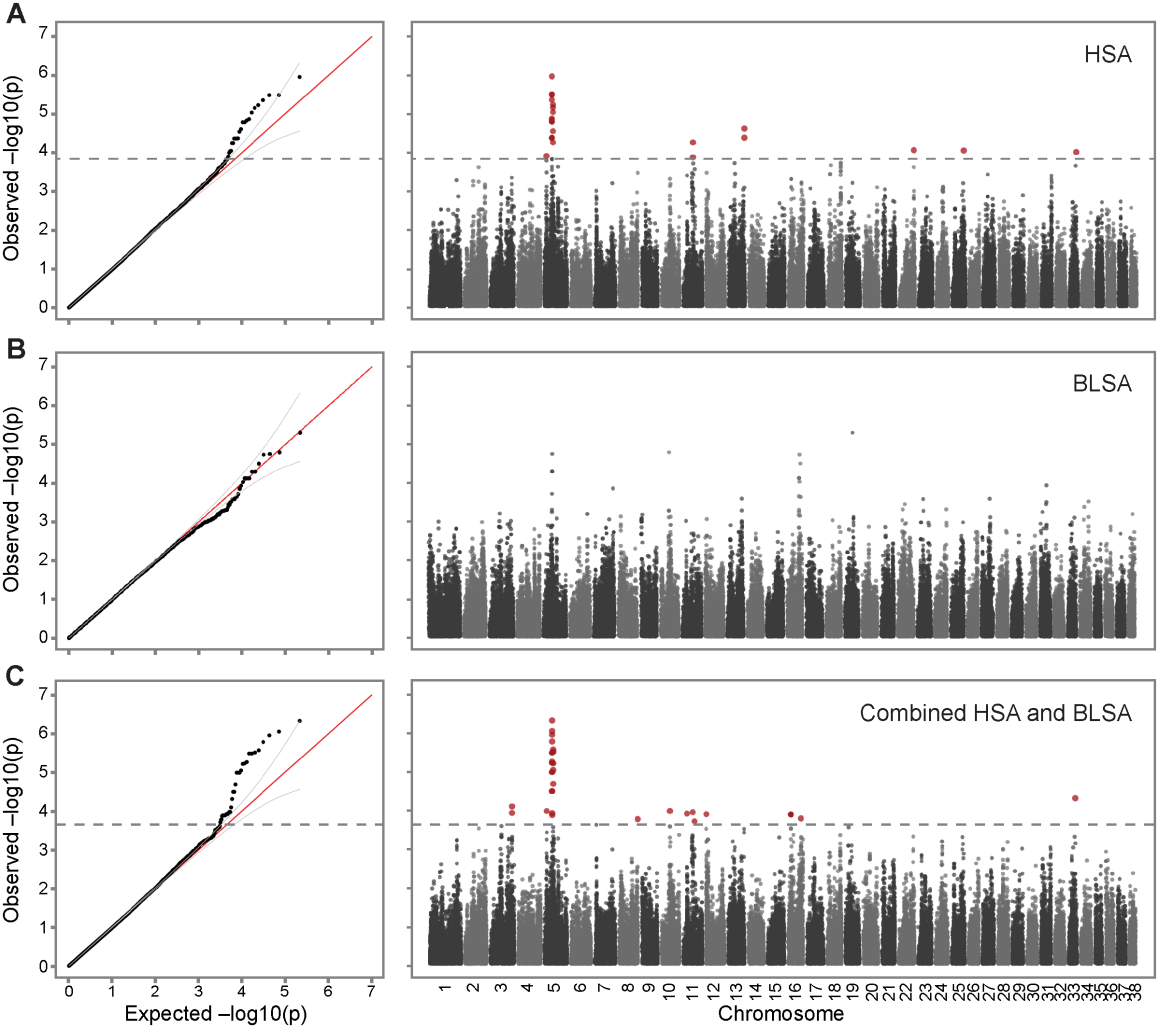
Genome-wide association of hemangiosarcoma and B-cell lymphoma identifies chromosome 5 as a common risk factor. A. Association of 142 cases with hemangiosarcoma and 172 healthy controls. The inflation factor λ of this analysis is 0.959, indicating that the population stratification had been properly controlled. The observed p-values deviated from the null beyond 95% confidence interval at-logP = 3.84, with a strong peak on chromosome 5, and a few SNPs on other chromosomes reaching significance. B. Analysis of 41 B-cell lymphoma cases and 172 healthy controls (λ = 0.976). C. As both lymphoma and hemangiosarcoma were most strongly associated to the same region on chromosome 5, the datasets were combined (142 hemangiosarcoma + 41 B-cell lymphoma cases and 172 controls) and reanalyzed for association, resulting in an increased association signal on chromosome 5 at p-value of 4.63 × 10^−7^ (λ = 0.988, significance threshold-logP = 3.66). Sex and the first PC was used as covariates in all association studies.

To search for inherited risk factors predisposing to hemangiosarcoma in golden retrievers, we performed GWAS by genotyping 148 hemangiosarcoma cases and 172 cancer-free golden retrievers >10 years old using the canineHD Illumina 170k SNP array [23]. Since dog breeds contain high levels of cryptic relatedness and complex family structures, it was necessary to apply a method to control for the population stratification [24] (Methods), and a final dataset of 142 hemangiosarcoma cases and 172 controls, and 108,973 SNPs was used for the association analysis. The quantile-quantile plot (QQ-plot) showed an inflation factor λ of 0.959, indicating that the population stratification had been well controlled (Fig. 1A). SNPs with p-values below 1.45×10^−4^ significantly deviate from the expected distribution, and as the Manhattan plot of p-values estimated by GCTA [25] shows, the main association signal comes from chromosome 5, with other less significantly associated peaks on chromosomes 11 and 13 (Fig. 1A). For the chromosome 5 peak, the top SNP (regression odds ratio (OR_regres_) = 1.23, p-value = 1.09×10^−6^) was located at 29,892,306 bp, 85 kb upstream of *TRPC6* and in strong LD (r^2^ > 0.8) with 10 other significantly associated SNPs (Table 1). The four most associated SNPs are all in high LD with each other. The next three significantly associated SNPs are all located within the STX8 gene, around 33.8–34.1 Mb; two more significantly associated SNPs are in LD with SNPs at 33 Mb (Table 1).

**Table 1:** List of significantly associated SNPs from each GWAS.

**Hemangiosarcoma, significantly associated SNPs**										
SNP	Chr	Position (bp)	alleles (minor/major)	MAF (cases)	MAF (controls)	P	Odds Ratio (regr.)	r^2^ from 29 Mb top SNP (BICF2G63035726)	r^2^ from 33Mb-shared top SNP (BICF2G630183630)	r^2^ from 33Mb-BLSA top SNP (BICF2G630183623)
BICF2S23035109	5	8756081	G/A	0.30	0.17	1.27E-04	1.19	n/a	n/a	n/a
BICF2G63035476	5	29699676	G/A	0.31	0.49	1.48E-05	1.20	0.88	0.09	0.02
BICF2S23317145	5	29716926	G/A	0.31	0.49	1.36E-05	1.20	0.88	0.09	0.02
BICF2P1405079	5	29748609	T/G	0.30	0.47	4.32E-05	1.19	0.83	0.09	0.01
BICF2G63035510	5	29748871	C/A	0.30	0.47	4.32E-05	1.19	0.83	0.09	0.01
BICF2G63035542	5	29762601	C/T	0.30	0.47	4.32E-05	1.19	0.83	0.09	0.01
BICF2G63035564	5	29778962	A/G	0.30	0.48	1.64E-05	1.20	0.85	0.09	0.02
BICF2G63035577	5	29795750	C/T	0.30	0.48	1.64E-05	1.20	0.85	0.09	0.02
BICF2G63035700	5	29867304	G/T	0.28	0.47	4.38E-06	1.21	0.98	0.09	0.02
BICF2G63035705	5	29870177	G/T	0.27	0.47	3.23E-06	1.22	0.99	0.09	0.02
BICF2G63035726	5	29892306	C/T	0.27	0.48	1.09E-06	1.23	<top>	0.09	0.02
BICF2G63035729	5	29893423	C/T	0.27	0.47	3.23E-06	1.22	0.99	0.09	0.02
BICF2G630183626	5	33851492	C/T	0.21	0.09	2.87E-05	1.28	0.09	0.99	0.36
BICF2G630183630	5	33854327	C/T	0.22	0.09	7.00E-06	1.30	0.09	<top>	0.37
BICF2G630183805	5	34088493	A/G	0.21	0.10	5.64E-05	1.26	0.09	0.95	0.34
BICF2P267306	5	34106119	A/G	0.23	0.10	9.21E-06	1.29	0.09	0.87	0.31
BICF2P1337948	5	34117726	G/T	0.23	0.10	5.91E-06	1.30	0.09	0.88	0.33
BICF2P22260	11	37603913	A/T	0.27	0.15	1.37E-04	1.21	n/a	n/a	n/a
BICF2P858820	11	37765641	T/C	0.18	0.07	5.65E-05	1.28	n/a	n/a	n/a
BICF2P1362415	13	61519478	T/C	0.13	0.03	2.46E-05	1.36	n/a	n/a	n/a
BICF2G630746301	13	61533573	C/T	0.12	0.03	4.27E-05	1.36	n/a	n/a	n/a
BICF2S23119401	22	56864599	C/A	0.33	0.23	8.95E-05	1.20	n/a	n/a	n/a
BICF2G630105651	25	44610750	T/C	0.40	0.57	9.14E-05	1.16	n/a	n/a	n/a
TIGRP2P389582	33	25358942	C/T	0.27	0.17	1.00E-04	1.21	n/a	n/a	n/a
**B-cell lymphoma, top chr 5 SNPs (not significantly associated)**										
SNP	Chr	Position (bp)	alleles (minor/major)	MAF (cases)	MAF (controls)	P	Odds Ratio (regr.)	r^2^ from 29 Mb top SNP (BICF2G63035726)	r^2^ from 33Mb-shared top SNP (BICF2G630183630)	r^2^ from 33Mb-BLSA top SNP (BICF2G630183623)
BICF2G630183354	5	33422865	A/G	0.24	0.04	6.48E-05	1.36	0.05	0.51	0.91
BICF2G630183623	5	33845636	T/C	0.23	0.04	2.34E-05	1.39	0.05	0.58	<top>
BICF2G630183652	5	33888351	C/T	0.22	0.04	6.39E-05	1.37	0.06	0.56	0.97
**Combined hemangiosarcoma + B-cell lymphoma, significantly associated SNPs**										
SNP	Chr	Position (bp)	alleles (minor/major)	MAF (cases)	MAF (controls)	P	Odds Ratio (regr.)	r^2^ from 29 Mb top SNP (BICF2G63035726)	r^2^ from 33Mb-shared top SNP (BICF2G630183630)	r^2^ from 33Mb-BLSA top SNP (BICF2G630183623)
TIGRP2P54568	3	83457175	G/T	0.45	0.31	1.19E-04	1.17	n/a	n/a	n/a
BICF2P678427	3	83463617	A/G	0.45	0.31	8.05E-05	1.17	n/a	n/a	n/a
BICF2S23035109	5	8756081	G/A	0.30	0.17	1.06E-04	1.19	n/a	n/a	n/a
BICF2G63035383	5	29613573	T/A	0.32	0.48	3.2E-05	1.18	0.73	0.09	0.03
BICF2G63035403	5	29623349	T/C	0.32	0.48	3.2E-05	1.18	0.73	0.09	0.03
BICF2G63035476	5	29699676	G/A	0.32	0.49	5.89E-06	1.19	0.87	0.10	0.03
BICF2S23317145	5	29716926	G/A	0.32	0.49	5.44E-06	1.19	0.87	0.10	0.03
BICF2P1405079	5	29748609	T/G	0.30	0.47	1.03E-05	1.19	0.81	0.10	0.03
BICF2G63035510	5	29748871	C/A	0.30	0.47	1.03E-05	1.19	0.81	0.10	0.03
BICF2G63035542	5	29762601	C/T	0.30	0.47	1.03E-05	1.19	0.81	0.10	0.03
BICF2G63035564	5	29778962	A/G	0.30	0.48	3.23E-06	1.20	0.82	0.10	0.03
BICF2G63035577	5	29795750	C/T	0.30	0.48	3.23E-06	1.20	0.82	0.10	0.03
BICF2G63035700	5	29867304	G/T	0.28	0.47	1.09E-06	1.21	0.97	0.10	0.04
BICF2G63035705	5	29870177	G/T	0.28	0.47	8.74E-07	1.22	0.98	0.10	0.03
BICF2G63035726	5	29892306	C/T	0.29	0.48	4.63E-07	1.22	<top>	0.10	0.03
BICF2G63035729	5	29893423	C/T	0.29	0.47	1.62E-06	1.21	0.99	0.10	0.03
BICF2P93507	5	30000139	A/G	0.49	0.34	1.19E-04	1.17	0.44	0.00	0.04
BICF2P342766	5	30313603	A/G	0.36	0.26	1.34E-04	1.18	0.27	0.34	0.18
BICF2G630183626	5	33851492	C/T	0.22	0.09	9.02E-06	1.27	0.09	0.99	0.45
BICF2G630183630	5	33854327	C/T	0.23	0.09	2.66E-06	1.28	0.10	<top>	0.45
BICF2G630183805	5	34088493	A/G	0.22	0.10	2.07E-05	1.25	0.10	0.96	0.44
BICF2P267306	5	34106119	A/G	0.24	0.10	6.07E-06	1.26	0.10	0.88	0.41
BICF2P1337948	5	34117726	G/T	0.25	0.10	3.05E-06	1.27	0.10	0.89	0.42
BICF2S22951928	8	72096016	C/T	0.35	0.47	1.71E-04	1.15	n/a	n/a	n/a
BICF2G630484859	10	32674924	T/C	0.32	0.48	1.05E-04	1.17	n/a	n/a	n/a
BICF2G630293768	11	14869362	C/T	0.19	0.11	1.23E-04	1.23	n/a	n/a	n/a
BICF2P858820	11	37765641	T/C	0.17	0.07	1.13E-04	1.25	n/a	n/a	n/a
BICF2P1128413	11	44451759	G/T	0.09	0.04	1.95E-04	1.32	n/a	n/a	n/a
BICF2P858293	12	1213363	C/T	0.35	0.49	1.27E-04	1.16	n/a	n/a	n/a
BICF2P1265909	16	10974488	G/A	0.05	0.14	1.29E-04	1.28	n/a	n/a	n/a
BICF2P1305119	16	10979219	T/C	0.05	0.14	1.29E-04	1.28	n/a	n/a	n/a
BICF2P1334089	16	10986902	T/C	0.05	0.14	1.29E-04	1.28	n/a	n/a	n/a
BICF2P51352	16	11005128	C/T	0.05	0.14	1.29E-04	1.28	n/a	n/a	n/a
BICF2G630817643	16	50321589	T/C	0.48	0.36	1.64E-04	1.17	n/a	n/a	n/a
TIGRP2P389582	33	25358942	C/T	0.27	0.17	4.89E-05	1.21	n/a	n/a	n/a

A separate GWAS for B-cell lymphoma in golden retrievers was performed using 41 cases and the same 172 controls as for the hemangiosarcoma study. Since the case sample size was relatively small, stricter cutoffs were used to control for population stratification, but due to careful selection of controls based on pedigrees, all of the 41 cases and 172 controls, and 109,579 SNPs remained in the dataset for the association analysis. The QQ-plot revealed that although no SNPs reach genome-wide significance for this small dataset of cases, there are three SNPs with p-values below 1×10^−4^ that deviate from the null distribution. These three SNPs are located on chromosome 5 at 33.4–33.9 Mb, and have OR_regres_ of 1.36–1.39.

### Combined GWAS identifies shared risk loci

The hemangiosarcoma dataset showed a strong association on chromosome 5. The B-cell lymphoma signal was considerably weaker and no SNP reached genome-wide significance, but the association signals overlapped with the hemangiosarcoma signal on chromosome 5. Therefore, we combined the datasets to assess if the two diseases had common predisposing risk factors. After quality and relatedness control, 183 cases (142 hemangiosarcoma cases and 41 B-cell lymphoma cases), 172 controls, and 109,407 SNPs were analyzed for the association. The QQ plot deviated from the null distribution at 2.2×10^−4^, identifying 35 significantly associated SNPs (best p-value = 4.63×10^−7^, Fig. 1C, Table 1), of which 20 were located on chromosome 5 between 29.6 Mb and 34.1 Mb. Sixteen SNPs out of these 20 SNPs were identical to the significantly associated SNPs from the hemangiosarcoma analysis, all of them with more significant p-values in the combined study, confirming their importance in B-cell lymphoma. The associated SNPs in this region clustered in two peaks located 4 Mb apart. The top SNPs in the two regions were located at 29,892,306 bp and 33,854,327 bp, with p-value of 4.63×10^−7^ and 2.66×10^−6^, respectively.

**Figure 2:**
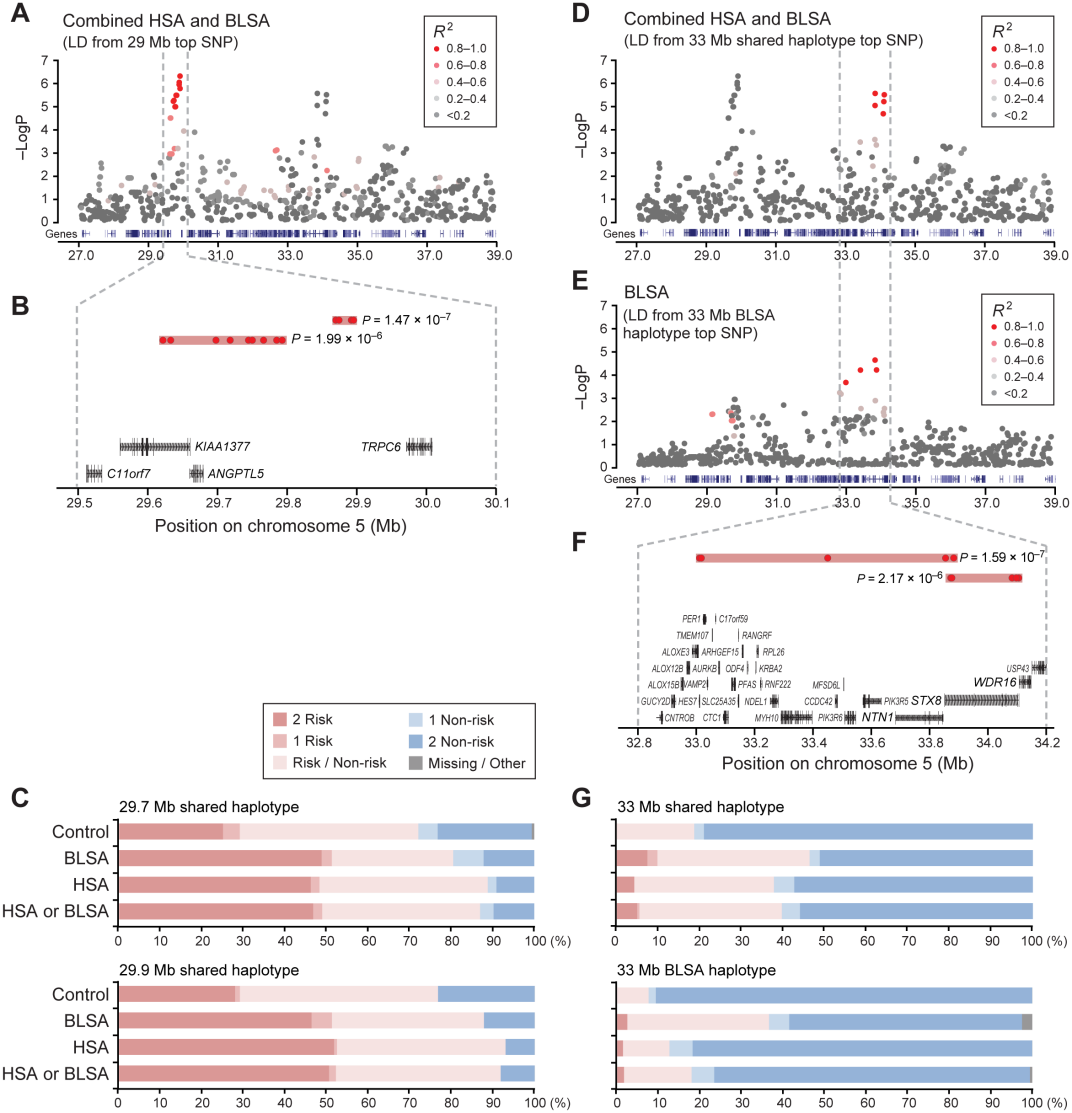
Two neighboring loci on chromosome 5 are independently associated with disease risk. A. The top SNP of the first peak (29 Mb) is in high LD with nearby variants and shows no evidence of linkage to the top SNPs in the second peak (33 Mb). B. The 29 Mb peak is comprised of two haplotype blocks, and C. the risk haplotypes for the 29 Mb peak are rather common in the population. Similarly, D. the second peak also shows no linkage with the first peak in the combined analysis, whereas E. analysis of only B-cell lymphoma shows SNPs in strong LD within the second peak and in moderate LD with SNPs in the first peak. The top SNPs in the combined analysis and B-cell-lymphoma-only analysis are independent, and F. make up separate haplotypes at the second locus. G. Both risk haplotypes at the second locus are rare. Color-coding of SNPs in A, D, E, reflects their r^2^ value relative the top SNP of that region, ranging from grey (not in LD) to red (strong LD).

Importantly, the two loci located 4 Mb apart were tagging different risk haplotypes. For the combined dataset, the top SNP in each region shows high LD (r^2^ > 0.8) with SNPs within the same peak, but low LD (r^2^ < 0.2) to the associated SNPs in the other peak (Table 1, Fig. 2 A, D, S1–S3 Fig.). To further confirm that these loci are not in linkage, we conducted conditional association analyses, which included the genotype of the top SNP of one peak as a covariate (Methods), and the results also indicate that the two peaks are independent signals (S1–S3 Fig.). Detailed analyses of the associated risk haplotypes in the separate and combined datasets shows that the 29 Mb risk alleles are mostly predicting hemangiosarcoma predisposition, although the association is stronger in the combined dataset compared to hemangiosarcoma alone. The 33 Mb region is associated with disease in both datasets, and interestingly, the top SNPs differ in the hemangiosarcoma and combined, vs the B-cell lymphoma dataset (Table 1, Fig. 2D, E). The respective top SNP from each analysis, located 8.7 kb apart, are in high LD (r^2^>0.8) with several SNPs around them, but not with each other (r^2^ = 0.45, combined dataset). They are tagging two different haplotypes in the 33 Mb region. SNPs in the B-cell lymphoma risk haplotype are not significantly associated with hemangiosarcoma (Table 1) and p-values drop in the combined analysis compared to B-cell lymphoma alone, suggesting that this is an independent haplotype only predisposing to B-cell lymphoma. The SNPs of these two haplotypes are interspersed along the genome (S1 Table).

### Risk haplotypes are common at one locus, rare at the other

To define the exact risk haplotypes and their boundaries, r^2^-based clumping analysis was performed by PLINK [26, 27], and r^2^-based block definition and association analysis was performed by Haploview [28] (Methods). These analyses identified risk and non-risk haplotypes in both loci. In the 29 Mb region two associated haplotype blocks were seen: a 9-SNP block (“29.7Mb-shared”) spanning 182 Kb, and a 4-SNP block (“29.9Mb-shared”) spanning 26 kb (Table 2, Fig. 2). The risk haplotypes largely appear in the same dogs, suggesting the possibility of selection in this region (S2A Table). In the 33 Mb region, a 5-SNP haplotype block (“33Mb-shared”) spanning 266 kb was identified in the combined dataset (Table 2, Fig. 2, S1 Table). An additional, B-cell-lymphoma-specific haplotype was identified at 33 Mb (“33Mb-BLSA”), which consists of 4 SNPs spanning over 887 kb. An r^2^-based haplotype analysis of the chromosome 5 region including both peaks using the combined dataset showed no long-range haplotype spanning two peaks, thus further confirming the independence of these two peaks. Notably, the BLSA-33Mb risk haplotype is in LD (r^2^ = 0.75) with 4 SNPs in the 29 Mb region (Fig. 2E). Those SNPs are interspersed with the top SNPs at 29 Mb identified in the combined analysis.

**Table 2:** List of significantly associated haplotypes.

**Hemangiosarcoma**	**Haplotype**	**Frequency (case, control)**	**ChiSq**	**Allelic OR**	**P (raw)**	P (empirical) *
29.7Mb-shared	ACAAGATGT	(0.68,0.49)	21.55	2.12	3.45E-06	3.10E-06
29.9Mb-shared	TTTT	(0.72,0.53)	25.68	2.37	4.02E-07	**6.00E-07**
33Mb-shared	CCAAG	(0.21,0.09)	17.47	2.73	2.92E-05	2.23E-05
33Mb-BLSA	GCTA	(0.07,0.04)	3.60	1.85	5.79E-02	8.25E-02
**B-cell lymphoma**						
29.7Mb-shared	ACAAGATGT	(0.65,0.49)	6.26	1.82	1.24E-02	**1.32E-02**
29.9Mb-shared	TTTT	(0.69,0.53)	6.826	1.83	8.98E-03	**1.92E-02**
33Mb-shared	CCAAG	(0.28,0.09)	18.91	3.94	1.37E-05	**1.87E-05**
33Mb-BLSA	GCTA	(0.21,0.04)	27.48	6.85	1.59E-07	**5.40E-06**
**Combined**						
29.7Mb-shared	ACAAGATGT	(0.67,0.49)	22.78	2.02	1.81E-06	**2.60E-06**
29.9Mb-shared	TTTT	(0.72,0.53)	27.08	2.22	1.95E-07	**3.00E-07**
33Mb-shared	CCAAG	(0.22,0.09)	22.75	2.91	1.85E-06	1.60E-06
33Mb-BLSA	GCTA	(0.10,0.04)	10.78	2.68	1.03E-03	**1.30E-03**

*P-value generated by permutation test with 10^7^ iterations. Significant p-values are shown in bold. Haplotypes that are not significantly associated in one disease are listed for comparison purposes.

The risk haplotypes at the 29 Mb locus have a high frequency (Fig. 2C, S3 Table); almost half of all cases are homozygous for the risk haplotype as compared to 25% in the control dogs for the 29.7Mb-shared risk haplotype. The frequencies are similar for the 29.9Mb-shared haplotype. For both haplotypes, the percentage of dogs homozygous for the risk allele is considerably larger among the cases compared to controls (S3 Table).

In contrast, the risk haplotypes at the 33 Mb locus have a much lower frequency; only 7% in dogs with B-cell lymphoma and 4% in dogs with hemangiosarcoma are homozygous risk, while about a third are heterozygous for the 33Mb-shared risk haplotype. In comparison, not a single control dog is homozygous risk, and one in five are heterozygous for this risk haplotype (Fig. 2, S3 Table). The disparate frequency of the risk alleles at the two loci also supports a hypothesis of two distinct risk factors. The separate B-cell lymphoma risk haplotype (33Mb-BLSA) is also rare; 2% of B-cell lymphoma and 1% of hemangiosarcoma cases are homozygous for this haplotype and 34% and 11%, respectively, are heterozygous. In contrast, no control dog is homozygous for the risk haplotype and 8% are heterozygous for the risk haplotype. The 33Mb-BLSA risk haplotype appears to be tagging a newer variant that occurred on the existing, shared risk haplotype. Every 33Mb-BLSA risk allele is carried with a 33Mb-shared risk allele, such that dogs homozygous for the 33Mb-BLSA risk haplotype are also homozygous for the 33Mb-shared risk haplotype, and all dogs heterozygous for the 33Mb-BLSA risk haplotype have at least one copy of the 33Mb-shared risk haplotype. This is a significant deviation from what would be expected if the two haplotypes were unlinked (p_ChiSq_ = 7.3×10^−50^) (S2A Table).

To determine the proportion of disease risk explained by the genotypes of these two loci, we performed a restricted maximum likelihood (REML) analysis using GCTA software [25] (Methods). All the autosomes together explain 43.2% ± 17.1% of the phenotype (p-value = 5.6 × 10^−4^), and the SNPs within 25–40 Mb on chromosome 5 explain 22.4% ± 10.7% (p-value = 2.7 × 10^−5^) of the phenotype in the combined analysis (S4 Table). These results suggest that the two risk loci on chromosome 5 account for ~20% of the phenotypic variance of these cancers in the golden retriever breed.

### Chromosome 5 germ-line risk factors influence expression of genes important in immune responses

Two approaches were taken to evaluate potential candidate genes within the regions of association. In summary, no protein-coding changes associated with either risk or non-risk haplotypes were found, but the risk haplotypes at both loci had a strong effect on the expression level of genes that play important roles in the immune response, especially T-cell mediated responses.

Specifically, we first examined the coding exons of genes within the most strongly associated regions for risk-haplotype-concordant non-synonymous germ-line mutations using ~40x coverage of Illumina sequence from nine individuals (Methods). At the 29 Mb locus, *KIAA1377* harbored two SNPs that would lead to amino acid substitutions if they were translated but they are likely intronic, *ANGPTL5* has one coding mutation, and *TRPC6* has two mutations in the 5’ UTR (S5 Table). For *NTN1, STX8*, and *WDR16*, genes near the 33 Mb locus, one non-synonymous mutation was found in *WDR16* and two in NTN1 (S5 Table). However, none of those mutations was associated with the risk haplotype while deviating from the mammalian consensus.

Secondly, since no coding changes were identified, we investigated whether the risk haplotypes were associated with transcriptional changes in tumors. We generated RNA-Seq data from 22 hemangiosarcoma and 22 B-cell lymphoma samples. The gene expression in the hemangiosarcoma samples reflected their high levels of contamination by stroma cells, which is typical for hemangiosarcoma tumors, and no conclusions could be drawn. The B-cell lymphoma samples were more homogeneous, and were grouped into “higher-risk” and “lower-risk” categories depending by how many copies of the risk allele they possessed.

**Figure 3:**
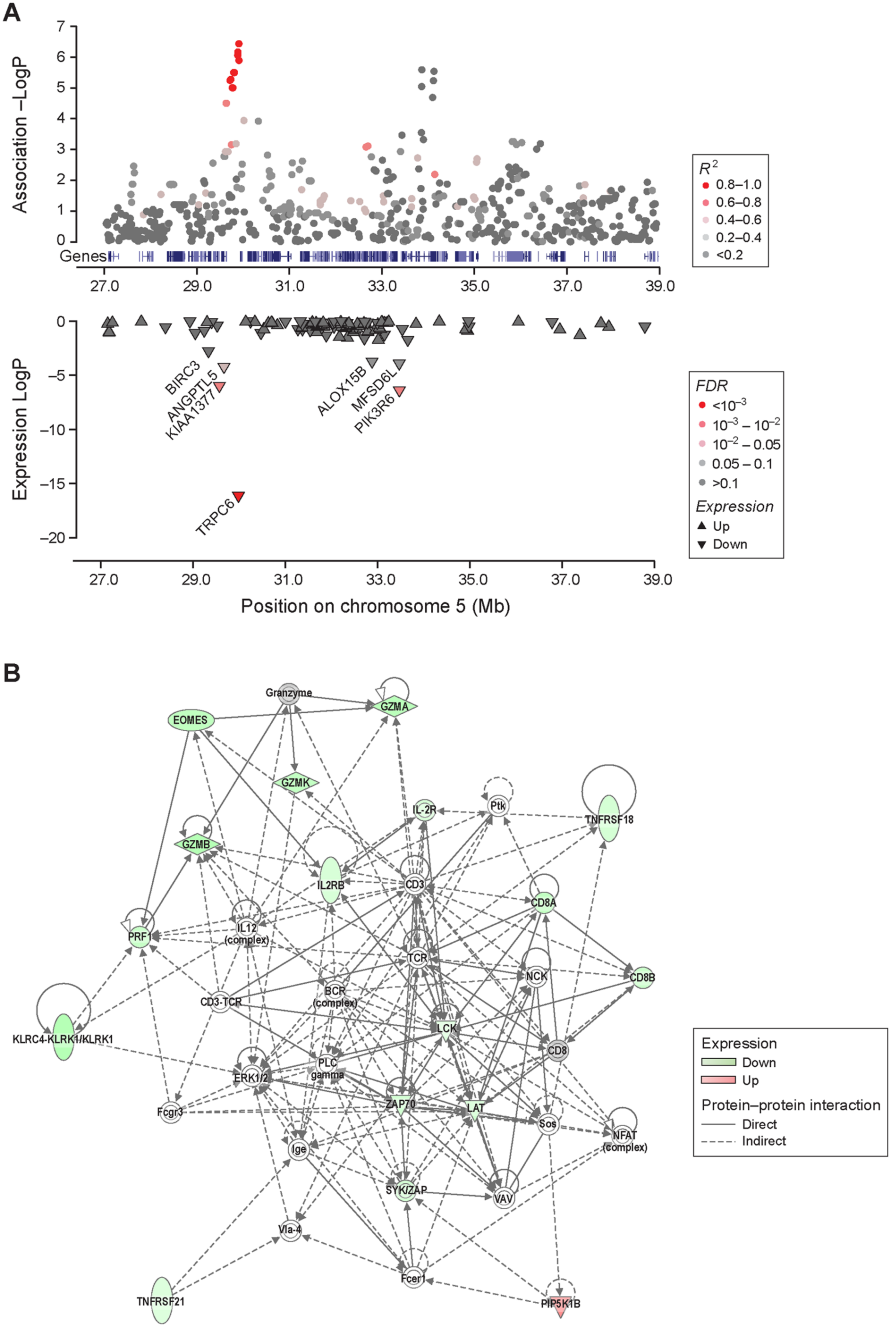
Differentially expressed genes by the risk alleles at 29 Mb and 33 Mb play important role in T-cell immunity. A. The risk allele at the 29 Mb at homozygous state has a clear cis-regulation effect on the expression levels of *TRPC6*, *KIAA1377*, and *ANGPTL5*, three of the most proximal genes. *BIRC3*, which is also proximal to the 29 Mb risk locus, had a significant p-value, however the FDR value was slightly above the threshold of 0.05. The risk allele at 29 Mb was also associated with a regulatory effect on genes near the 33 Mb locus and a change in the expression of *PIK3R6* significantly. B. A large network of molecules that play a major role in activation of T-lymphocyte and other immune cells (IPA category: cell-to-cell signaling and interaction, hematological system development and function). This network includes 15 molecules of which expressions are significantly altered in individuals carrying at least one copy of the shared risk allele at the 33 Mb locus. The outcomes of such expression changes are significantly linked to decrease in T-cell activation.

Briefly, for the 29 Mb locus, 12 dogs homozygous for the risk haplotype were designated as the higher-risk group and compared to the lower-risk group consisting of mostly heterozygous dogs (eight heterozygous dogs and two dogs with no copy of the risk haplotype). The same individuals were higher-risk or lower-risk for both 29.7Mb-shared and 29.9Mb-shared haplotypes. The results show that the risk haplotype at 29 Mb had a clear cis-regulatory effect (Fig. 3A, Table 3, S6 Table), and most significantly altered the expression of *TRPC6*, the closest gene to 29.9Mb-shared (logFC_risk_ = −7.46, p-value = 7.45 × 10^−17^, FDR = 1.37 × 10^−12^, Table 3, Fig. 3A). The expression of the *TRPC6* transcript was virtually undetectable in the tumors of dogs in the higher-risk group (all dogs are homozygous for the risk haplotype). *TRPC6* encodes a transient receptor potential channel, which mediates calcium ion (Ca^2+^) influx [29] and plays a significant role in T-cell activation through at least two pathways; 1) the PLCγ pathway regulated by the T-cell receptor, and 2) the PI3K pathway that is mediated by co-stimulation through CD28 [30, 31].

**Table 3:** Top 10 differentially expressed genes by the risk haplotype at each locus.

**29 Mb risk analysis**					
**Gene Name**	logFC_risk_*	**p-value**	**FDR**	**Chr**	**Start first exon**
TRPC6	-7.46	7.45E-17	1.37E-12	5	29,974,951
FGFR4	-4.37	1.46E-07	8.96E-04	4	36,241,080
RPL6	1.74	2.78E-07	1.28E-03	26	9,970,456
PIK3R6	-1.69	3.88E-07	1.43E-03	5	33,471,196
GFRA2	-3.72	9.26E-07	2.62E-03	25	35,437,179
KIAA1377	-2.54	1.01E-06	2.62E-03	5	29,559,277
SCARA5	-2.68	1.13E-06	2.62E-03	25	29,591,101
GRM5	-3.04	1.48E-06	2.90E-03	21	10,982,218
FABP4	-3.4	1.57E-06	2.90E-03	X	2,439,049
U2	5.65	4.55E-06	7.00E-03	4	12,989,141
**33 Mb risk analysis (shared haplotype)**					
Gene Name	logFC_risk_ *	p-value	FDR	Chr	Start first exon
IGLV2–33	5.98	5.36E-12	9.89E-08	26	27,164,804
CD5L	-3.46	3.84E-07	3.12E-03	7	40,515,318
CXCL10	-3.37	6.55E-07	3.12E-03	32	597,634
SLC25A48	-4.65	6.76E-07	3.12E-03	11	23,754,003
KRT24	-5.79	1.76E-06	5.50E-03	9	21,967,227
HIST1H4L	2.92	2.62E-06	6.91E-03	17	59,129,682
IGHV3–64	-3.62	5.29E-06	1.14E-02	8	73,477,706
GPR27	-3.91	5.57E-06	1.14E-02	20	20,271,644
GZMA	-2.82	8.06E-06	1.31E-02	2	42,490,492
HS3ST3B1	1.96	9.27E-06	1.31E-02	5	38,023,684

*Fold change was calculated by designating the lower-risk group as a reference.

For the 33 Mb locus, a higher-risk group of mostly heterozygous dogs (one homozygous and five heterozygous for the 33Mb-shared risk haplotype) were compared to the lower-risk group of 16 dogs carrying no copy of the 33Mb-shared risk haplotype (Methods). Five of the six higher-risk dogs carried the 33Mb-BLSA risk haplotype, which is consistent with the genotyping data where all dogs carrying the 33Mb-BLSA risk haplotype also carry the 33Mb-shared risk haplotype (S2B Table). Having at least one copy of the 33Mb-shared risk haplotype at 33 Mb significantly changed the expression levels of 100 genes located elsewhere in the genome (Table 3, S6 Table). None of the 100 genes were within 1 Mb of any of the significantly associated loci in either the hemangiosarcoma, B-cell lymphoma, or combined GWAS. Unsupervised clustering (S4 Fig.) did not group the samples relating to their haplotypes, suggesting that the differential gene expression associated with the risk haplotypes is not the key differentiator of tumors. A knowledge based Ingenuity Pathway Analysis (IPA)[32] of the 100 genes based on the 33Mb-shared haplotype identified a large number of common biological functions including differentiation, activation and cell-to-cell signaling in the immune system (S7 Table). The 33Mb-shared risk allele was shown to mediate overall decreases in immune cell activation (Fig. 3B, S7 Table). Eighteen significant canonical pathways were identified (S8 Table), and of the top four pathways (p-value < 0.005) three directly implicate T-cell responses. Several upstream regulators, including IL-2 (z-score = −2.97, p-value = 5.62×10^−14^), CD3 (z-score = 2.02, p-value = 3.34×10^−13^), TCR (z-score = −2.83, p-value = 6.31×10^−13^), ZBTB7B (z-score = 2.21, p-value = 1.13×10^−9^) and IL-15 (z-score = −2.63, p-value = 2.96×10^−9^) were identified, all of which play an important role in the activation, acquisition of effector functions and lineage differentiation of T-cells [33–35] (S9 Table).

## Discussion

GWAS of human DLBCL using thousands of human patients have detected a few candidate loci, which together only account for a small fraction of the genetic risk [12, 14, 15]. For human angiosarcoma, no GWAS has been performed due to the rarity of the disease. Here we performed GWAS for canine B-cell lymphoma and hemangiosarcoma using fewer than 400 dogs for both diseases combined, and identified two loci of strong effect accounting for about 20% of the disease risk. This study illustrates the advantages of mapping a complex trait within a canine breed, in which a small number of risk factors with a strong effect are present as a result of the strong bottlenecks at breed creation, and the relative genetic homogeneity within the breed. The fact that one of the two risk factors on chromosome 5 (29 Mb) is very common in the U.S. golden retriever population may relate to the use of popular sires. It also could be an example of a strong genetic risk factor accumulating either through drift or selective breeding for a nearby locus.

It was unexpected and remarkable to discover that two rather different cancers, B-cell lymphoma and hemangiosarcoma are linked to the same inherited risk factors, as shown by the increased strength of association when combining the two datasets. While surprising, this could be explained by previous observations that hemangioblasts have the ability to generate both hematopoietic stem cells and endothelial cells [36], and that canine hemangiosarcoma is likely to originate from hemangioblasts [37]. Another remarkable finding is that only two loci appear to explain 20% of the total disease risk. This may be partly due to the homogenous genetic background present within this dog breed, but may also result from the effect size of the individual risk factors.

While the risk loci on chromosome 5 explain as much as 20% of the risk, no coding mutations were identified. Instead, we found that the risk haplotypes of both loci are significantly associated with gene expression changes, implying that the mutations in regulatory regions play an important role in cancer, which is often the case in other common diseases [38]. Several candidate loci fall just above or below the significance threshold in our current analyses. Since all autosomes together can explain an additional ~21% of the risk, incorporation of additional cases and controls in the future will likely identify more risk loci with genome-wide significance. In this context we note that the 41 B-cell lymphoma cases alone produced a relatively weaker signal for the chromosome 5 locus at 29 Mb, suggesting that for this high-frequency risk allele at OR_allelic_ ~2.0, a higher sample number would be needed to reach genome-wide significance, as our original power calculations predicted that at least 100 cases and 100 controls are required for mapping such alleles at less than 4% false positive rate with 80% power [20].

We find the existence of at least four disease-associated haplotypes in the two nearby chromosome 5 regions intriguing, and speculate that there may be genes in the region affecting traits for which dogs are bred in this population. In small, inbred populations like dog breeds, one popular individual can have many offspring, allowing certain haplotypes to become relatively common.

We note that no coding changes agree with the risk haplotypes, suggesting the presence of regulatory mutations. To identify the actual causative mutations additional bioinformatics analysis, validation genotyping in a larger sample set and functional analysis of key candidate variants will likely be necessary. It will also be useful to survey the frequency of the risk haplotypes in different golden retriever populations, for example those from the US and Europe where disease frequencies are reported to vary.

RNA-Seq data from B-cell lymphomas demonstrated an almost complete reduction of *TRPC6* transcript suggesting cis-regulation by the 29 Mb risk haplotype, which also reduced the expression of three other genes in the region *BIRC3, ANGPTL5*, and *KIAA1377*. *BIRC3* encodes an anti-apoptotic protein associated with B-cell malignancies and other cancers [39], ANGPTL5 is a member of the angiopoietin growth factor family [40], while KIAA1377 is a novel centrosomal protein required for cytokinesis [41]. *TRPC6* encodes a transient receptor potential channel, which mediates calcium ion (Ca^2+^) influx [29]. Interestingly, *TRPC6* is not normally expressed in B-cells [42], but has been reported to play an important role in T-cell activation [30, 43]. The expression levels of *TRPC6* have been shown to significantly alter levels of intracellular Ca^2+^ elevation and T-cell activation, which are mediated by at least two pathways; the PLCγ pathway regulated by the T-cell receptor, and the PI3K pathway that is mediated by co-stimulation through CD28 [30, 31]. Notably, the 33 Mb risk allele also suppressed the expression levels of many genes that are involved in the activation of immune responses, particularly T-cell activation. The regulation from the 33 Mb region appears to be trans-regulatory, but the exact mechanism to elicit this effect is unknown at present. One possibility is that a cis-regulatory effect of the risk haplotype on an undiscovered lincRNA in this region could be mediating the trans-regulatory effect. The different effects of the combined risk haplotype and the B-cell lymphoma specific haplotype at this locus cannot be distinguished without further work. Notably, several of the suggested top upstream regulators of the 100 genes affected by the 33Mb haplotype are possible targets of NF-κB [44], which could suggest that the effect of the risk haplotype could be mediated by pathways affected by NF-κB. Because of the altered gene expression, we hypothesize that the germ-line mutations tagged by the risk haplotypes in the associated loci lead to T-cell dysfunction that plays an important role in B-cell lymphoma and hemangiosarcoma development.

The expression levels of T-cell markers, such as CD28 and CD3 epsilon, were not affected by the risk haplotypes, so the expression reduction in *TRPC6* and other genes involved in T-cell activation was not due to the absence of T-cells within the tumor. We also did not observe any expression differences in markers for NK cells and dendritic cells, such as CD3 zeta, CD11b, CD11c, CD56, and CD68. This is important to note, as the expression levels of certain chemotaxins and receptors, including *CCL5*, *CCL19*, *CCL22*, and *CCR6*, which attract lymphocytes, macrophages and/or dendritic cells [45–47] were decreased in dogs carrying the 33Mb-shared risk haplotype. In previous studies, different quantities of these cells in B-cell lymphoma have been linked to diagnostic and prognostic significance in humans as well as dogs [48–55].

In conclusion, we have identified two loci explaining ~20% of the risk for both hemangiosarcoma and B-cell lymphoma in US golden retrievers. While the discovery of the mutation(s) and the related mechanisms that lead to tumorigenesis is dependent on future studies, this study demonstrates the power of dogs for mapping germ-line risk factors with strong relevance for human cancer, as well as the importance of non-coding inherited risk factors in cancer predisposition. The strong correlation between the germ-line risk haplotypes and the expression changes that are indicative of immune dysfunction generates a novel hypothesis of how germ-line risk factors contribute to tumorigenesis. This novel hypothesis warrants further investigations both in canine and human lymphoma and angiosarcoma.

## Methods

### Study participants and inclusion criteria

All of the golden retrievers in the study were recruited from the privately owned pet population in the US. The owner voluntarily agreed to participate in the study, and a signed consent form was obtained for each participant. All the work described is in accordance to ethical guidelines and is included in the ethical approval protocols on “canine research”, MIT CAC 0910–074–13 (Lindblad-Toh). Diagnosis of B-cell lymphoma was confirmed by histological examination of the tumor as well as by PARR assay [56]. Diagnosis of hemangiosarcoma was obtained by one or more of the following methods: histological examination of formalin fixed tumor tissue, examination of cell surface markers by flow-cytometry, and by the pathology reports that were submitted by the dog owner or their veterinarian, which confirmed hemangiosarcoma diagnosis. Some of the hemangiosarcoma cases that had acute and extensive abdominal hemorrhage with an ultrasound report of multiple cavitated and blood-filled tumors in more than one organ, and those having the characteristic right atrial tumor were included in the study without histological confirmation. Controls were confirmed to be cancer-free by owner questionnaire at the point of sample submission, and by periodic health updates. The age when a dog was last confirmed as healthy was used to determine inclusion. All control dogs’ pedigrees were carefully checked before picking dogs for genotyping to avoid introducing stratification. Cases’ pedigrees were also checked to avoid including closely related individuals when possible.

### GWAS analysis

Genomic DNA was isolated from whole blood and was genotyped for 170,000 SNPs using the Illumina 170K canine HD array [23] at the Broad Institute of MIT and Harvard, or at GeneSeek Inc (Lincoln, NE). To successfully control for the population stratification present in the dataset, we took an analysis approach based on a method described by Price et al. [24] First, the genome-wide SNP dataset was analyzed by PLINK [27, 57] (PLINK1.9 was used whenever possible, otherwise PLINK1.07) to apply standard quality filters including genotyping rate per SNP (>95%) and per individual (>95%), and minor allele frequency (MAF, >5%). Chromosome X was excluded because of the risk of it not being handled correctly in mixed model genetic relatedness calculations. Secondly GCTA [25] was used to estimate a genetic relationships matrix (grm) to remove excessively related individuals, and to calculate the principal components of the whole-genome SNP genotype data per individual by the EIGENSTRAT method [58], which was used as a covariate in the final step. Finally, GCTA [25] was used to test for the disease-genotype association with adjustment for the IBS matrix and for the first principal component, both calculated by GCTA. The threshold for genome-wide significance for each association analysis was defined based on the 95% confidence intervals (CIs) calculated from the beta distribution of observed p values, a method adopted from the study by the Wellcome Trust Case Control consortium [59]. Sex was used as a covariate. For the conditional analysis to address the independence of the two peaks on chromosome 5, the genotype of a top SNP of one peak/haplotype was used as the first covariate and sex was used as the second covariate.

For the GWAS of hemangiosarcoma, we genotyped 148 hemangiosarcoma cases (107 histologically confirmed cases, and 41 presumed cases including 16 with tumor in the right atrium of the heart), and 172 healthy controls > 10 years of age. After quality control and removal of excessively related individuals (grm value > 0.75), the final dataset analyzed for the hemangiosarcoma association included 142 cases, 172 controls and 108,973 SNPs. For the GWAS of B-cell lymphoma, we genotyped 41 histologically confirmed B-cell lymphoma cases and they were compared to the 172 healthy controls used for the analysis of hemangiosarcoma. To control for population stratification in this small dataset, grm value of 0.25 was used as the cut-off to remove dogs related at greater than the half-sibling level within the cases, and in the controls. After the filtering, the final dataset analyzed for the B-cell lymphoma association included 41 cases, 172 controls and 109,579 SNPs. For the combined analysis, after quality control and removal of excessively related individuals (grm value > 0.75), the final dataset analyzed for the association included 183 cases (142 hemangiosarcoma cases and 41 B-cell lymphoma cases), 172 controls, and 109,407 SNPs. We further independently validated the genotypes of the 24 top SNPs in a subset of 250 dogs by Sequenom (miscalling rate 0.0038).

### Haplotype block definition, and association analysis

The haplotype blocks in the associated loci were defined with boundaries that were commonly identified by the clumping analysis using PLINK [26, 27] and r^2^ based LD analysis by Haploview [28]. PLINK clumping analysis was performed by setting parameters as follow: association p-value for the index SNP < 1 × 10^−4^, r^2^ > 0.8 or 0.9, and a physical distance limit of 1 Mb. The Haploview analysis was performed by calculating pair-wise r^2^ values for the SNPs between 28 Mb and 36 Mb on chromosome 5 with a 2 Mb distance limit, and haplotype blocks were defined by r^2^ > 0.8 or 0.9. The haplotype blocks commonly identified by both analyses were used for further analysis. Haplotypes of each block, their allelic frequencies, chi-square test, allelic odds ratio and p-values (P_raw_) were obtained using PLINK. Each haplotype was then tested for association significance by running a permuted chi-square test for 10^7^ iterations using PLINK.

### Restricted maximum likelihood (REML) analysis

Estimation of the phenotypic variance explained by genetic variance was performed by REML analysis using GCTA [60], following online instructions on the GCTA website. In our analyses, the variance of the genetic factor was determined by the genotypes of SNPs on all autosomes, on each autosome separately, and within the associated region (25–40 Mb) on chromosome 5. Sex was used as a covariate. The estimate of variance explained on the observed scale is transformed to that on the underlying scale by the estimated disease prevalence of the general population. A p-value for each analysis is calculated based by performing a log-likelihood ratio test. We estimated prevalence as 0.20 for hemangiosarcoma, 0.0625 for B-cell lymphoma [5], and 0.2625 for being affected by either cancer, as it is extremely rare for one dog to have both cancers.

### Whole genome sequencing and analysis

Whole-genome paired-end sequencing was performed for germ-line DNA from nine golden retrievers, of which six were from the GWAS cohort. For each sample, approximately 1 billion 101 base-pair paired-end reads at 40x coverage were generated using Illumina HiSeq 2000. Picard pipeline [61] was used for data quality filtering and alignment of the reads to the canFam3.1 reference genome. The Genome Analysis Toolkit’s (GATK’s) UnifiedGenotyper [62] was then used to make genotype calls from the cleaned alignments. The resulting variants were then annotated based on the conservation across species using SEQscoring [63, 64], annotated and analyzed for predicted effect by using snpEff [65], and were visually examined by IGV [66] to look for variants likely to cause biological changes, and that are concordant with the disease-associated haplotypes. One variant was evaluated with SIFT [67].

### RNA sequencing and expression analysis

Twenty-two canine nodal B-cell lymphoma and twenty-two hemangiosarcoma samples (one tumor sample per dog) were analyzed by high-density RNA sequencing (20 million paired end reads). Total RNA was isolated from a whole frozen naïve (untreated) tumor tissue or cryopreserved single cell suspension of naïve tumor cells. Indexed Illumina sequencing libraries were constructed, size selected to 320 bp +/- 5%, and 50 base-pair paired-end reads were generated by Illumina HiSeq 2000. To estimate the abundance of different genes expressed in our samples, we first aligned the read data to canFam3.1 using TopHat [68] v1.4.1. The mate inner distance was set to 100 bp, and the maximum intron length was set to 500,000 bp. We then used HTSeq [69] v0.5.3p9 set for non-strand-specific data to perform read counting on genes. For a gene annotation, we used the canFam3.1 annotation supplemented with RNAseq data [70]. The expression levels were compared using edgeR [71] v3.0.8 to examine the relative gene expression changes associated with the presence or absence of approximately one copy of the risk haplotypes at 29 Mb or 33 Mb locus in the tumors. Given the high frequency of the risk allele, the 29 Mb “higher-risk” and “lower-risk” groups were defined as follows: a higher-risk group containing 12 dogs homozygous for risk haplotype; and a 29 Mb lower-risk group containing eight heterozygous dogs and two dogs with no copy of the risk haplotype (all dogs haplotypes were identical for the 29.7Mb-shared and 29.9-shared Mb). Because very few dogs were homozygous for the risk haplotype at the 33 Mb, the 33 Mb higher-risk and lower-risk groups were defined as follows: a higher-risk group of six dogs (five heterozygous and one homozygous for the 33Mb-shared risk haplotype); and a lower-risk group of 16 dogs with no copy of the risk haplotype. The groups were largely the same if defined from the 33Mb-BLSA risk haplotype, but the shared haplotype was used for group definition to be consistent with hemangiosarcoma analysis. B-cell lymphoma RNA was isolated from either tumor cells in suspension, or from a tumor biopsy that contained more stromal tissue (lymphocyte content > 90%, of those 85–100% were malignant cells). This known variable was applied as a blocking factor in edgeR analysis to reduce its influence in detecting the differences in gene expression. Expression differences between the groups with p-value and false discovery rate (FDR) of less than 0.05 were considered significant findings. Unsupervised clustering was performed using normalized FPKM values for the annotated genes, calculated for each sample using CuffNorm from Cufflinks 2.2.1. These values were then used as a feature vector and the dendrogram was created using the R v2.15 functions “dist” and “hclust”.

### Ingenuity Pathway Analysis

A knowledge-based functional analyses of the significant expression changes by the 29 Mb risk allele in 27 genes, and by the 33 Mb risk allele in 100 genes were performed by Ingenuity Pathway Analysis (IPA) [32]. Of the 27 and 100 genes examined, IPA mapped 25 and 89 genes respectively. The parameters for the core analysis were set to consider direct and indirect relationships of genes and endogenous chemicals at predicted and experimentally observed confidence levels. The p-values for the downstream functions and canonical pathway analyses were corrected for multiple testing by the Benjamini-Hochberg procedure, and resulting p-values less than 0.05 were considered significant. When the analysis of downstream functions or upstream regulators identified a gene set with “bias” in the direction of expression changes, significance was determined by the combination of a p-value of less than 0.05 and an activation z-score of less than-2.00 or greater than 2.00, following Ingenuity Systems’ recommendation. False discovery rate (FDR) cutoff was set to 0.05 and fold change (FC) cutoffs were 1 and-1 (in log2).

### Statistical analysis

All the p-values reported in this study were obtained by using the programs mentioned in each analysis method. Briefly, the p-values in GWAS analysis were obtained by using GCTA, with a mixed model approach to account for population stratification, and a 0–1 quantitative response variable to represent the case-control status. The significance of the slope coefficient of a SNP, which represents the effect size of the SNP is calculated by the standard *t* test based on the variance of the slope coefficients of the study cohort [72]. For case-control data, Haploview utilizes a simple chi-square test to calculate the phenotype-haplotype association p-values (P_raw_) [28], and the association significance p-value (P_perm_) was obtained as the empirical probability of observing chi-square values in permutation tests that exceeded the best observed chi-square value using PLINK1.07. The p-values obtained by edgeR to identify differentially expressed genes were calculated by fitting gene-wise generalized linear models, and then conducting likelihood ratio tests for the risk haplotype [71]. The p-values by IPA for the canonical pathways and downstream biological functions were calculated using Fisher’s Exact Test, comparing the proportion of genes from the provided list mapping to a function or pathway to the proportion genes in the IPA database in that function or pathway [32]. The p-values were then corrected for multiple testing by the Benjamini-Hochberg procedure [32]. The upstream regulator analysis calculates the “overlap p-values” using Fisher’s Exact Test, which measures whether there is a statistically significant overlap between the observed gene set and the genes that are regulated by a particular transcriptional regulator [32].

### Data access

GWAS data are available on the Broad Institute’s website (www.broadinstitute.org/ftp/pub/vgb/dog/HSA_BLSA_PlosGenetics2014_paper/). WGS and RNA-Seq data are available via the NCBI BioProject site (WGS: PRJNA247491, RNA-Seq: PRJNA267721-267742).

## Supporting Information

S1 Fig LD between the two neighboring loci on chromosome 5 for hemangiosarcoma analysis and conditional association analyses for the top SNPs reveal that the two neighboring loci are independent. **A**. r^2^ values were calculated from the top SNP at 29 Mb to other SNPs in the region, or **B**. r^2^ values were calculated from the top SNP at 33 Mb to other SNPs in the region, and the coloring reflects r^2^ values, ranging from grey (not in LD) to red (strong LD). In this study cohort, the top SNPs in these two peaks are not in LD (r^2^ < 0.2). **C**. r^2^ values were calculated from the top SNP in the B-cell lymphoma specific haplotype at 33 Mb. SNP. In order to test if the two loci are showing independent association signals, each association analysis was performed with a primary covariate that represents the genotypes of **D**. the top SNP at 29 Mb, **E**. the top SNP at 33 Mb (33Mb-shared haplotype), and **F**. the top SNP at 33 Mb (33Mb-BLSA haplotype). Concordant with the LD structure observations, the association signal of a peak was still detected even with the conditioning on the top SNP of the other peak, indicating independent association. Sex was used as covariate in all association studies (secondary covariate in the conditional analysis).
(TIF)

S2 Fig LD between the two neighboring loci on chromosome 5 for B-cell lymphoma analysis and conditional association analyses for the top SNPs reveal that the two neighboring loci are independent. The two loci on chromosome 5 detected in hemangiosarcoma had stronger association when the B-cell lymphoma cases were added, although they didn’t reach genome-wide significance in this dataset alone. Even though it was not significant each locus had a separate peak, therefore, to test if they were independent loci in the B-cell lymphoma dataset, **A**. r^2^ values were calculated from the top SNP of the combined analysis at 29 Mb to other SNPs in the region, or **B**. r^2^ values were calculated from the top SNP of the combined analysis at 33 Mb to other SNPs in the region. In this study cohort, the top SNPs in these two peaks are not in LD (r^2^ < 0.2). **C**. r^2^ values were calculated from the top SNP in the B-cell lymphoma predisposing haplotype at 33 Mb. SNP coloring reflects r^2^ value, ranging from grey (not in LD) to red (strong LD). In order to test if the two loci are showing independent association signals, each association analysis was performed with a primary covariate that represents the genotypes of **D**. the top SNP at 29 Mb, **E**. the top SNP at 33 Mb (33Mb-shared haplotype), and **F**. the top SNP at 33 Mb (33Mb-BLSA haplotype). Concordant with the LD structure observations, the association signal of a peak was still detected even with the conditioning on the top SNP of the other peak, indicating independent association. Sex was used as covariate in all association studies (secondary covariate in the conditional analysis).
(TIF)

S3 Fig LD between the two neighboring loci on chromosome 5 in the combined dataset and conditional association analyses for the top SNPs reveal that the two neighboring loci are independent. To test if the identified loci on chromosome 5 were independent loci in the combined dataset, **A**. r^2^ values were calculated from the top SNP at 29 Mb to other SNPs in the region, or **B**. r^2^ values were calculated from the top SNP at 33 Mb to other SNPs in the region, and the coloring reflects r^2^ value, ranging from grey (not in LD) to red (strong LD). In this study cohort, the top SNPs in these two peaks are not in LD (r^2^ < 0.2). **C**. r^2^ values were calculated from the top SNP in the B-cell lymphoma specific haplotype at 33 Mb. SNP. In order to test if the two loci are showing independent association signals, each association analysis was performed with a primary covariate that represents he genotypes of **D**. the top SNP at 29 Mb, **E**. the top SNP at 33 Mb (33Mb-shared haplotype), and **F**. the top SNP at 33 Mb (33Mb-BLSA haplotype). Concordant with the LD structure observations, the association signal of a peak was still detected even with the conditioning on the top SNP of the other peak, indicating independent association. Sex was used as covariate in all association studies (secondary covariate in the conditional analysis).
(TIF)

S4 Fig Unsupervised clustering of RNA-Seq samples does not form groups related to the differential expression seen in high-risk and low-risk groups. The RNA source (0, tissue or 1, cells), and the grouping into high- and low-risk for the two loci (H, high-risk and L, low-risk) are indicated. RNA source was corrected for in analysis.
(TIF)

S1 Table Haplotype block definitions. Position (canFam3.1) and ID of SNPs constituting the four identified haplotypes.
(PDF)

S2 Table Coexistence of risk haplotypes at 29 and 33 Mb. Number of observed individuals and their haplotypes (R, risk; a, alternative) at the **A**. 29 Mb locus or **B**. 33 Mb locus.
(PDF)

S3 Table Frequency of risk haplotypes. Frequency of individuals being homozygous risk, heterozygous risk, or homozygous non-risk for each haplotype in the respective datasets.
(PDF)

S4 Table Variance explained by chromosome 5 or all autosomes, as estimated by REML. Variance explained with and without sex as covariate in the respective datasets.
(PDF)

S5 Table List of germ-line non-synonymous mutations in genes at the 29 and 33 loci. Non-synonymous mutations in exons including 5’ UTR.
(PDF)

S6 Table Differentially expressed genes by the risk haplotype at each locus. Genes differentially expressed in B-cell lymphomas when comparing tumors that are high-risk to low-risk at the 29 and 33 Mb loci.
(PDF)

S7 Table Significantly affected biological functions downstream of the observed gene expression changes by the 33 Mb risk haplotype. Biological functions predicted by IPA to be altered as a result of the differential gene expression seen in tumors that are high-risk at the 33 Mb locus.
(PDF)

S8 Table Canonical pathways with significant (p < 0.05) enrichment of the genes with expression changes by the 33 Mb risk haplotype. Canonical pathways estimated by IPA to be affected as a result of the differential gene expression seen in tumors that are high-risk at the 33 Mb locus.
(PDF)

S9 Table Upstream regulators of the observed gene expression changes by the 33 Mb risk haplotype. Upstream regulators suggested by IPA to explain the differential gene expression seen in tumors that are high-risk at the 33 Mb locus.
(PDF)
