# Correction: Genome-wide Association Study Identifies Shared Risk Loci Common to Two Malignancies in Golden Retrievers

**DOI:** 10.1371/journal.pgen.1005339

**Published:** 2015-06-24

**Authors:** The PLOS Genetics Staff

## Notice of Republication

This article was republished on May 26, 2015, in order to correct headings for Table 1 which were incorrect. The headings for Table 1 are now correct in the republished manuscript. Please download this article again to view the correct version. The originally published, uncorrected article and the republished, corrected article are provided here for reference.

## Supporting Information

S1 File Originally published, uncorrected article. (PDF)

S2 File Republished corrected article. (PDF)

## References

[1] Tonomura N, Elvers I, Thomas R, Megquier K, Turner-Maier J, Howald C, et al (2015) Genome-wide Association Study Identifies Shared Risk Loci Common to Two Malignancies in Golden Retrievers. PLoS Genet 11(2): e1004922 doi:10.1371/journal.pgen.1004922 25642983 10.1371/journal.pgen.1004922 PMC4333733

