# Supplementary figures and images for: Genome-wide Association Study Identifies Shared Risk Loci Common to Two Malignancies in Golden Retrievers
Source: PLoS Genet. 2015 Feb 2;11(2):e1004922. doi: 10.1371/journal.pgen.1004922 (PMC4333733; doi:10.1371/journal.pgen.1004922)

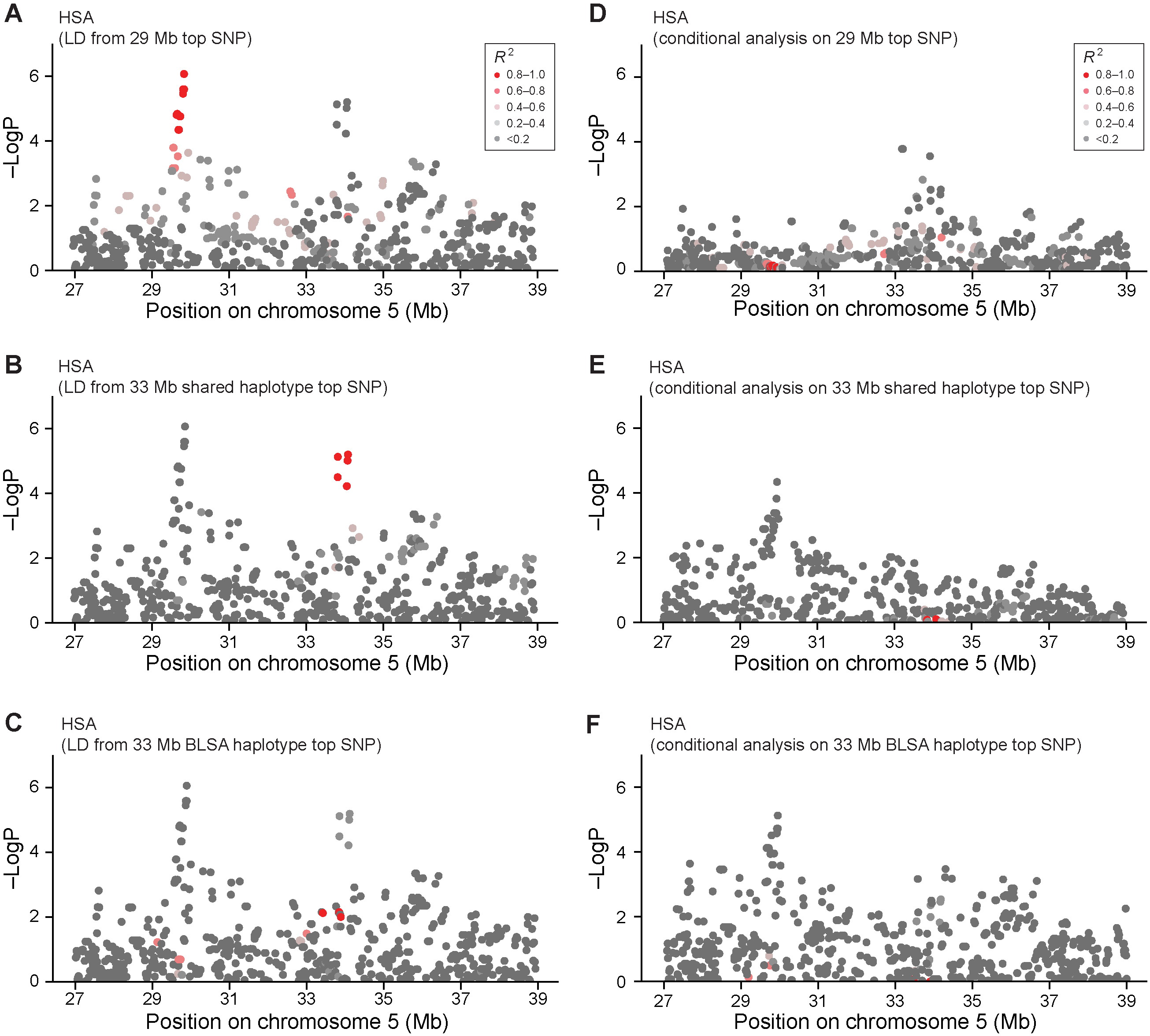

Supplement: S1 Fig — A. r2 values were calculated from the top SNP at 29 Mb to other SNPs in the region, or B. r2 values were calculated from the top SNP at 33 Mb to other SNPs in the region, and the coloring reflects r2 values, ranging from grey (not in LD) to red (strong LD). In this study cohort, the top SNPs in these two peaks are not in LD (r2 < 0.2). C. r2 values were calculated from the top SNP in the B-cell lymphoma specific haplotype at 33 Mb. SNP. In order to test if the two loci are showing independent association signals, each association analysis was performed with a primary covariate that represents the genotypes of D. the top SNP at 29 Mb, E. the top SNP at 33 Mb (33Mb-shared haplotype), and F. the top SNP at 33 Mb (33Mb-BLSA haplotype). Concordant with the LD structure observations, the association signal of a peak was still detected even with the conditioning on the top SNP of the other peak, indicating independent association. Sex was used as covariate in all association studies (secondary covariate in the conditional analysis). (TIF) [file pgen.1004922.s001.tif]

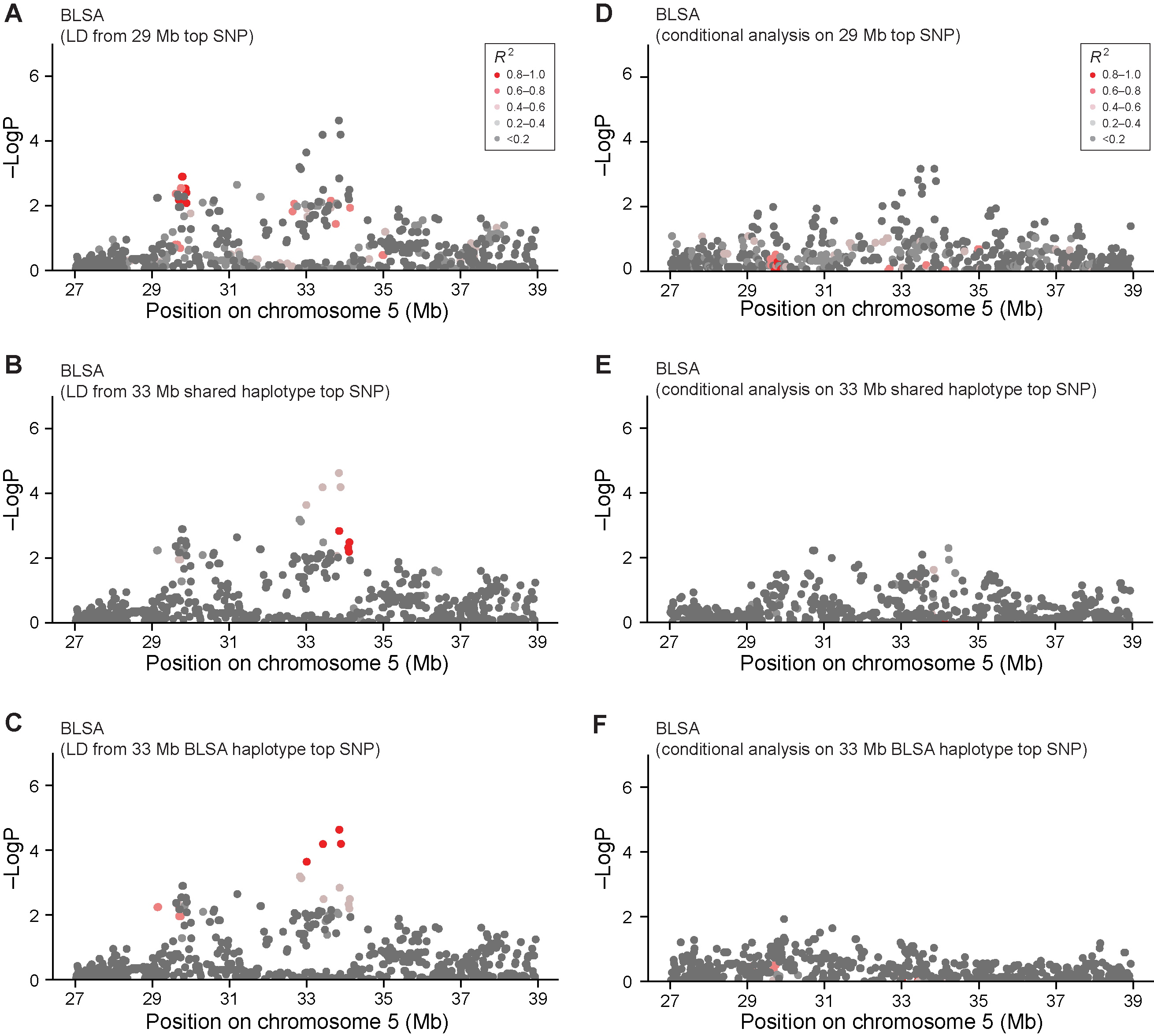

Supplement: S2 Fig — The two loci on chromosome 5 detected in hemangiosarcoma had stronger association when the B-cell lymphoma cases were added, although they didn’t reach genome-wide significance in this dataset alone. Even though it was not significant each locus had a separate peak, therefore, to test if they were independent loci in the B-cell lymphoma dataset, A. r2 values were calculated from the top SNP of the combined analysis at 29 Mb to other SNPs in the region, or B. r2 values were calculated from the top SNP of the combined analysis at 33 Mb to other SNPs in the region. In this study cohort, the top SNPs in these two peaks are not in LD (r2 < 0.2). C. r2 values were calculated from the top SNP in the B-cell lymphoma predisposing haplotype at 33 Mb. SNP coloring reflects r2 value, ranging from grey (not in LD) to red (strong LD). In order to test if the two loci are showing independent association signals, each association analysis was performed with a primary covariate that represents the genotypes of D. the top SNP at 29 Mb, E. the top SNP at 33 Mb (33Mb-shared haplotype), and F. the top SNP at 33 Mb (33Mb-BLSA haplotype). Concordant with the LD structure observations, the association signal of a peak was still detected even with the conditioning on the top SNP of the other peak, indicating independent association. Sex was used as covariate in all association studies (secondary covariate in the conditional analysis). (TIF) [file pgen.1004922.s002.tif]

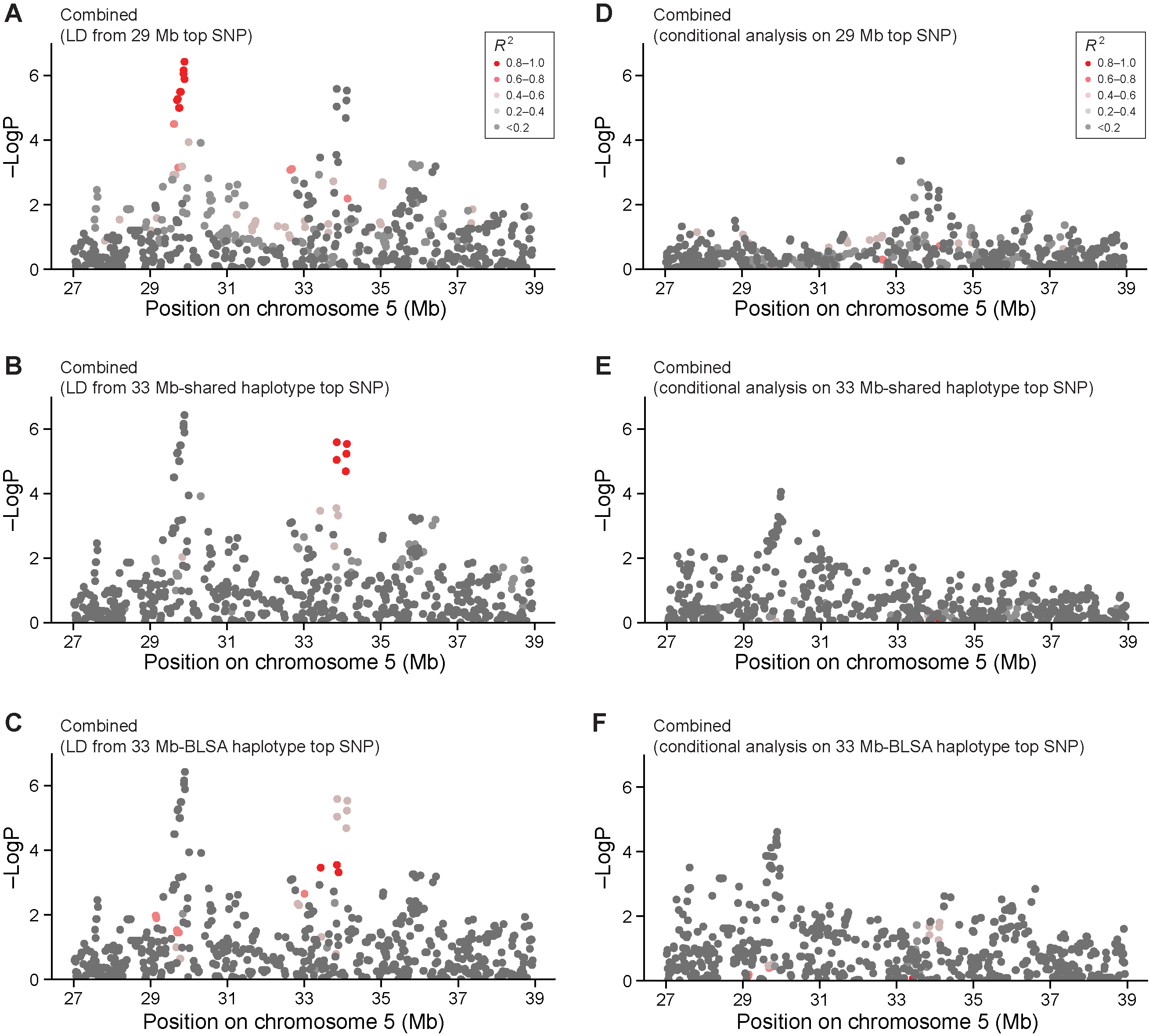

Supplement: S3 Fig — To test if the identified loci on chromosome 5 were independent loci in the combined dataset, A. r2 values were calculated from the top SNP at 29 Mb to other SNPs in the region, or B. r2 values were calculated from the top SNP at 33 Mb to other SNPs in the region, and the coloring reflects r2 value, ranging from grey (not in LD) to red (strong LD). In this study cohort, the top SNPs in these two peaks are not in LD (r2 < 0.2). C. r2 values were calculated from the top SNP in the B-cell lymphoma specific haplotype at 33 Mb. SNP. In order to test if the two loci are showing independent association signals, each association analysis was performed with a primary covariate that represents he genotypes of D. the top SNP at 29 Mb, E. the top SNP at 33 Mb (33Mb-shared haplotype), and F. the top SNP at 33 Mb (33Mb-BLSA haplotype). Concordant with the LD structure observations, the association signal of a peak was still detected even with the conditioning on the top SNP of the other peak, indicating independent association. Sex was used as covariate in all association studies (secondary covariate in the conditional analysis). (TIF) [file pgen.1004922.s003.tif]

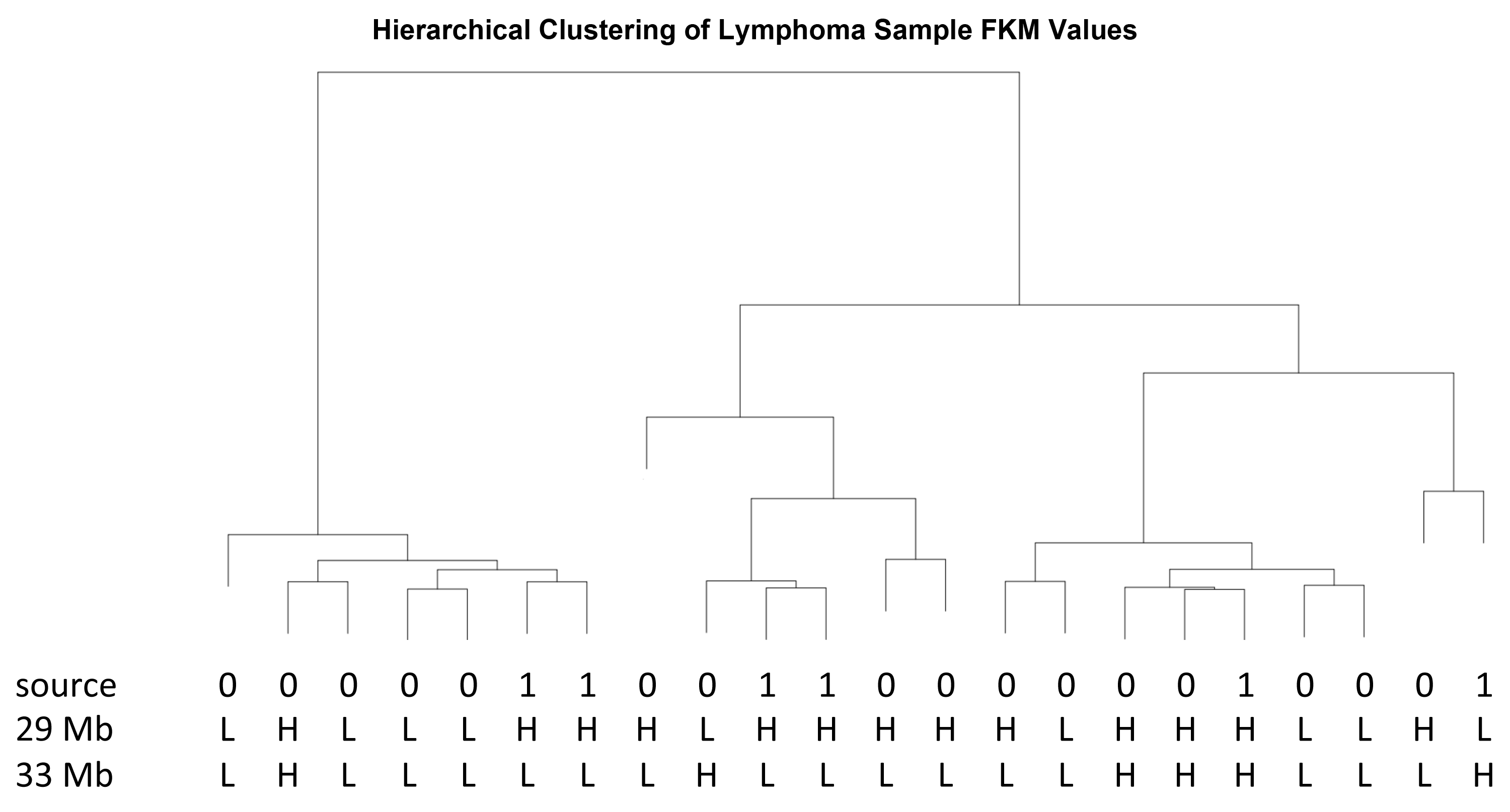

Supplement: S4 Fig — The RNA source (0, tissue or 1, cells), and the grouping into high- and low-risk for the two loci (H, high-risk and L, low-risk) are indicated. RNA source was corrected for in analysis. (TIF) [file pgen.1004922.s004.tif]
