# Supplementary material for: Genome-wide Association Study Identifies Shared Risk Loci Common to Two Malignancies in Golden Retrievers
Source: PLoS Genet. 2015 Feb 2;11(2):e1004922. doi: 10.1371/journal.pgen.1004922 (PMC4333733; doi:10.1371/journal.pgen.1004922)
Supplement: S1 Table — Position (canFam3.1) and ID of SNPs constituting the four identified haplotypes. (PDF) [file pgen.1004922.s005.pdf]

Supplementary Table 1. Haplotype block definitions

haplotype	SNPs	position (chr 5)
29.7Mb-shared haplotype	BICF2G63035383	29613573
	BICF2G63035403	29623349
	BICF2G63035476	29699676
	BICF2S23317145	29716926
	BICF2P1405079	29748609
	BICF2G63035510	29748871
	BICF2G63035542	29762601
	BICF2G63035564	29778962
	BICF2G63035577	29795750
29.9Mb-shared haplotype	BICF2G63035700	29867304
	BICF2G63035705	29870177
	BICF2G63035726	29892306
	BICF2G63035729	29893423
33Mb-shared haplotype	BICF2G630183626	33851492
	BICF2G630183630	33854327
	BICF2G630183805	34088493
	BICF2P267306	34106119
	BICF2P1337948	34117726
33Mb-BLSA haplotype	BICF2P639123	33001550
	BICF2G630183354	33422865
	BICF2G630183623	33845636
	BICF2G630183652	33888351
