# Supplementary material for: Genome-wide Association Study Identifies Shared Risk Loci Common to Two Malignancies in Golden Retrievers
Source: PLoS Genet. 2015 Feb 2;11(2):e1004922. doi: 10.1371/journal.pgen.1004922 (PMC4333733; doi:10.1371/journal.pgen.1004922)
Supplement: S2 Table — Number of observed individuals and their haplotypes (R, risk; a, alternative) at the A. 29 Mb locus or B. 33 Mb locus. (PDF) [file pgen.1004922.s006.pdf]

Supplementary Table 2. Coexistence of risk haplotypes at 29 and 33 Mb

A. 29 Mb

Observed individuals (excluding those with a recombination on either haplotype)

		29.7Mb-shared		
		RR	Ra	aa
29.9Mb-shared	RR	127	9	0
	Ra	0	134	9
	aa	0	0	48

R, risk. a, alternative (not risk)

B. 33 Mb

Observed individuals

		33Mb-shared		
		RR	Ra	aa
33Mb-BLSA	RR	3	0	0
	Ra	3	40	0
	aa	3	56	250

R, risk. a, alternative (not risk)
