# Supplementary material for: Genome-wide Association Study Identifies Shared Risk Loci Common to Two Malignancies in Golden Retrievers
Source: PLoS Genet. 2015 Feb 2;11(2):e1004922. doi: 10.1371/journal.pgen.1004922 (PMC4333733; doi:10.1371/journal.pgen.1004922)
Supplement: S3 Table — Frequency of individuals being homozygous risk, heterozygous risk, or homozygous non-risk for each haplotype in the respective datasets. (PDF) [file pgen.1004922.s007.pdf]

Supplementary Table 3. Frequency of risk haplotypes

29.7Mb-shared haplotype				
	# samples	Homozygous risk	Heterozygous risk	Homozygous non-risk
HSA	142	0.46	0.42	0.09
BLSA	41	0.49	0.32	0.12
combined cases	183	0.47	0.40	0.10
controls	172	0.25	0.47	0.23
29.9Mb-shared haplotype				
	# samples	Homozygous risk	Heterozygous risk	Homozygous non-risk
HSA	142	0.51	0.42	0.07
BLSA	41	0.46	0.41	0.12
combined cases	183	0.50	0.42	0.08
controls	172	0.28	0.49	0.23
33Mb-shared haplotype				
	# samples	Homozygous risk	Heterozygous risk	Homozygous non-risk
HSA	142	0.04	0.34	0.57
BLSA	41	0.07	0.39	0.51
combined cases	183	0.05	0.35	0.56
controls	172	0.00	0.19	0.79
33Mb-BLSA haplotype				
	# samples	Homozygous risk	Heterozygous risk	Homozygous non-risk
HSA	142	0.01	0.11	0.82
BLSA	41	0.02	0.34	0.56
combined cases	183	0.02	0.16	0.76
controls	172	0.00	0.08	0.91
