# Supplementary material for: Genome-wide Association Study Identifies Shared Risk Loci Common to Two Malignancies in Golden Retrievers
Source: PLoS Genet. 2015 Feb 2;11(2):e1004922. doi: 10.1371/journal.pgen.1004922 (PMC4333733; doi:10.1371/journal.pgen.1004922)
Supplement: S4 Table — Variance explained with and without sex as covariate in the respective datasets. (PDF) [file pgen.1004922.s008.pdf]

Supplementary Table 4. Variance explained by chromosome 5 or all autosomes, as estimated by REML

**Hemangiosarcoma**	*variance explained*	*SE*	*p*	*variance explained, sex as covariate*	*SE*	*p*
all autosomes	0.37	0.18	2.40E-03	0.40	0.18	1.32E-03
only chr 5	0.26	0.10	3.45E-04	0.25	0.10	2.91E-04
chr 5, 25-40 Mb	0.18	0.10	7.08E-05	0.18	0.10	4.23E-05
**B-cell lymphoma**						
all autosomes	1.11	0.48	6.55E-03	1.12	0.46	2.41E-03
only chr 5	0.22	0.18	7.72E-02	0.13	0.15	1.86E-01
chr 5, 25-40 Mb	0.63	0.25	1.66E-04	0.60	0.25	5.76E-04
**combined dataset**						
all autosomes	0.41	0.17	8.52E-04	0.43	0.17	6.58E-04
only chr 5	0.21	0.09	6.79E-04	0.19	0.09	1.19E-03
chr 5, 25-40 Mb	0.23	0.11	2.17E-05	0.22	0.11	2.76E-05
