# Supplementary material for: Genome-wide Association Study Identifies Shared Risk Loci Common to Two Malignancies in Golden Retrievers
Source: PLoS Genet. 2015 Feb 2;11(2):e1004922. doi: 10.1371/journal.pgen.1004922 (PMC4333733; doi:10.1371/journal.pgen.1004922)
Supplement: S5 Table — Non-synonymous mutations in exons including 5’ UTR. (PDF) [file pgen.1004922.s009.pdf]

Supplementary Table 5. List of germ-line non-synonymous mutations in genes at the 29 and 33 loci

Gene name	Non-synonymous change	CanFam3.1 position	Reason for disqualification as candidate
KIAA1377	C -> T, likely in intron	29,591,154	not concordant with any of the four risk haplotypes or with phenotype
	G -> A, likely in intron	29,660,431	not concordant with any of the four risk haplotypes or with phenotype
ANGPTL5	cAa (Q) -> cGa (R)	29,668,080	not concordant with any of the four risk haplotypes or with phenotype
TRPC6	A -> G, 5' UTR	29,975,006	not concordant with any of the four risk haplotypes or with phenotype
	C -> T, 5' UTR	29,975,035	not concordant with any of the four risk haplotypes or with phenotype
NTN1	gGc (G) -> gCc (A)	33,655,616	not concordant with any of the four risk haplotypes or with phenotype
	Tgc (C) -> Ggc (G)	33,655,720	not concordant with any of the four risk haplotypes or with phenotype
WDR16/CFAP52	Ggc (G) -> Agc (S)	34,121,952	Concordant with shared-33Mb haplotype, not with BLSA-33Mb haplotype. The amino acid change is predicted to be 'tolerated' by SIFT analysis. The risk allele is the allele that is conserved across mammals. SNP location 4.5 kb outside core risk haplotype.
