# Supplementary material for: Genome-wide Association Study Identifies Shared Risk Loci Common to Two Malignancies in Golden Retrievers
Source: PLoS Genet. 2015 Feb 2;11(2):e1004922. doi: 10.1371/journal.pgen.1004922 (PMC4333733; doi:10.1371/journal.pgen.1004922)
Supplement: S6 Table — Genes differentially expressed in B-cell lymphomas when comparing tumors that are high-risk to low-risk at the 29 and 33 Mb loci. (PDF) [file pgen.1004922.s010.pdf]

Supplementary Table 6. Differentially expressed genes by the risk haplotype at each locus

**29 Mb risk analysis**					
Gene Name	$logFC_{risk}$	p-value	FDR	Chr	Start first exon
TRPC6	-7.46	7.45E-17	1.37E-12	5	29,974,951
FGFR4	-4.37	1.46E-07	8.96E-04	4	36,241,080
RPL6	1.74	2.78E-07	1.28E-03	26	9,970,456
PIK3R6	-1.69	3.88E-07	1.43E-03	5	33,471,196
GFRA2	-3.72	9.26E-07	2.62E-03	25	35,437,179
KIAA1377	-2.54	1.01E-06	2.62E-03	5	29,559,277
SCARA5	-2.68	1.13E-06	2.62E-03	25	29,591,101
GRM5	-3.04	1.48E-06	2.90E-03	21	10,982,218
FABP4	-3.4	1.57E-06	2.90E-03	X	2,439,049
U2	5.65	4.55E-06	7.00E-03	4	12,989,141
CACNA1D	-2.74	5.11E-06	7.25E-03	20	36,191,810
MPO	3.69	7.28E-06	8.61E-03	9	32,924,779
GPC3	-3.9	7.39E-06	8.61E-03	X	104,313,240
HTR4	-3.07	7.47E-06	8.61E-03	4	60,275,413
KIF5C	-1.93	1.32E-05	1.43E-02	19	50,535,364
ZNF662	-2.43	1.52E-05	1.55E-02	23	11,979,368
KIAA1456	-2.01	2.52E-05	2.27E-02	16	36,462,321
COL6A6	-2.4	2.58E-05	2.27E-02	23	27,759,397
CD300C	1.51	3.06E-05	2.57E-02	9	5,906,878
NAA11	4.07	3.37E-05	2.63E-02	32	3,656,949
APM1	-4.1	3.43E-05	2.63E-02	34	19,398,225
RGS13	-5.92	4.69E-05	3.33E-02	38	6,288,032
ADAMTS2	-1.65	5.48E-05	3.74E-02	11	2,279,935

ANGPTL5	-3.1	5.73E-05	3.78E-02	5	29,659,261
AFF2	3.02	6.37E-05	4.02E-02	X	116,849,230
PCDH11Y	-2.51	6.53E-05	4.02E-02	X	68,639,234
MTUS1	-1.7	8.09E-05	4.82E-02	16	40,957,737

33 Mb risk analysis (shared haplotype)

Gene Name	$logFC_{risk}$	p-value	FDR	Chr	Start first exon
IGLV2-33	5.98	5.36E-12	9.89E-08	26	27,164,804
CD5L	-3.46	3.84E-07	3.12E-03	7	40,515,318
CXCL10	-3.37	6.55E-07	3.12E-03	32	597,634
SLC25A48	-4.65	6.76E-07	3.12E-03	11	23,754,003
KRT24	-5.79	1.76E-06	5.50E-03	9	21,967,227
HIST1H4L	2.92	2.62E-06	6.91E-03	17	59,129,682
IGHV3-64	-3.62	5.29E-06	1.14E-02	8	73,477,706
GPR27	-3.91	5.57E-06	1.14E-02	20	20,271,644
GZMA	-2.82	8.06E-06	1.31E-02	2	42,490,492
HS3ST3B1	1.96	9.27E-06	1.31E-02	5	38,023,684
CCR6	1.04	9.84E-06	1.31E-02	1	54,932,810
KIAA1598	-3.19	1.14E-05	1.35E-02	28	27,421,247
EXTL1	-3.45	1.17E-05	1.35E-02	2	73,932,214
PROK2	-5.98	1.38E-05	1.49E-02	20	20,249,470
KLRK1	-3	1.69E-05	1.65E-02	27	35,634,936
NPDC1	-1.08	1.93E-05	1.65E-02	9	48,596,282
OBSL1	-1.25	1.97E-05	1.65E-02	37	26,048,686
CNNM1	-8.14	1.98E-05	1.65E-02	28	12,289,827
LBH	-1.65	2.02E-05	1.65E-02	17	23,944,331
U6	1.93	2.51E-05	1.65E-02	36	4,076,401
IGHV3-66	-5.03	2.65E-05	1.65E-02	8	74,052,039

GALNT13	-9.26	2.69E-05	1.65E-02	36	642,898
STOM	-2	2.70E-05	1.65E-02	11	74,270,015
CSF1	-1.21	2.71E-05	1.65E-02	6	42,028,059
MMP25	-1.69	2.72E-05	1.65E-02	6	38,130,092
PTPN22	1.07	2.74E-05	1.65E-02	17	51,623,693
FAM190A	-2.26	2.77E-05	1.65E-02	32	13,499,039
PADI4	2.13	3.28E-05	1.89E-02	2	80,912,334
CD8A	-2.52	4.08E-05	2.22E-02	17	38,369,617
RAB19	-3.33	4.44E-05	2.34E-02	16	8,544,349
DLGAP3	2.95	5.69E-05	2.92E-02	15	7,058,384
EOMES	-2.56	6.02E-05	2.97E-02	23	16,505,623
MAPK11	-2.93	6.13E-05	2.97E-02	10	16,977,036
CCL22	-2.66	6.27E-05	2.97E-02	2	59,039,616
TMEM178	-3.12	6.73E-05	3.05E-02	17	31,633,521
HIST1H2AJ	1.14	6.77E-05	3.05E-02	35	24,155,213
CCDC168	1.98	7.45E-05	3.27E-02	22	52,228,513
TARM1	-2.44	8.11E-05	3.41E-02	1	103,180,872
NPTX2	-2.72	8.13E-05	3.41E-02	6	10,805,532
GZMK	-2.47	8.82E-05	3.44E-02	4	60,559,232
AFF2	-3.94	9.45E-05	3.44E-02	X	116,849,230
TRBV20OR9-2	-1.93	9.51E-05	3.44E-02	16	6,769,873
CD151	-0.92	1.01E-04	3.44E-02	18	45,195,481
TNFAIP3	-1.1	1.02E-04	3.44E-02	1	30,247,095
IGHV3-53	-2.92	1.05E-04	3.44E-02		unmapped
GZMB	-3.01	1.09E-04	3.44E-02	8	4,508,717
ACTL7A	-1.84	1.10E-04	3.44E-02	11	64,032,466
PLEKHG5	-1.5	1.12E-04	3.44E-02	5	60,320,850
IGLV1-50	-3.79	1.13E-04	3.44E-02	26	26,151,663
C17orf104	-3.1	1.15E-04	3.44E-02	9	18,795,120

FBXO11	1.67	1.16E-04	3.44E-02	10	49,839,202
MARCKSL1	-1.5	1.19E-04	3.44E-02	2	68,898,513
NKG7	-2.4	1.22E-04	3.44E-02	1	105,608,388
SMOC1	-3.07	1.22E-04	3.44E-02	8	43,608,321
ROR2	-3.32	1.25E-04	3.44E-02	1	95,235,633
CHRM4	-2.6	1.31E-04	3.44E-02	18	43,082,404
SCN2A	3.88	1.32E-04	3.44E-02	36	10,517,618
TBXA2R	-2.6	1.32E-04	3.44E-02	20	55,824,410
CCL5	-2.36	1.32E-04	3.44E-02	9	37,817,391
CCL19	-1.7	1.34E-04	3.44E-02	11	51,357,399
PIP5K1B	2.13	1.35E-04	3.44E-02	1	88,359,159
MT1	-2.19	1.36E-04	3.44E-02	2	59,602,961
IGLV3-9	-3.37	1.36E-04	3.44E-02	26	27,391,907
CORO6	-1.28	1.44E-04	3.58E-02	9	43,711,845
TLR10	0.87	1.66E-04	4.02E-02	3	73,568,321
TNIK	-1.81	1.68E-04	4.02E-02	34	35,427,908
PTH1R	-2.15	1.72E-04	4.06E-02	20	41,901,764
CHGA	-1.67	1.74E-04	4.06E-02	8	1,962,944
SLC38A11	1.83	1.83E-04	4.22E-02	36	10,118,571
COL17A1	-2.06	1.90E-04	4.29E-02	28	16,350,689
KEL	-1.43	1.92E-04	4.29E-02	16	6,626,235
CD8B	-2.13	1.93E-04	4.29E-02	17	38,342,989
TMPRSS13	3.53	1.98E-04	4.34E-02	5	15,725,911
TNFRSF21	-1.09	2.03E-04	4.37E-02	12	15,348,061
GSTA4	-1.02	2.05E-04	4.37E-02	12	20,426,726
TNFRSF4	-2.32	2.10E-04	4.37E-02	5	56,401,401
GRHL3	-3.47	2.10E-04	4.37E-02	2	75,290,529
ZAP70	-1.81	2.12E-04	4.37E-02	10	44,887,358
LAD1	-2.65	2.17E-04	4.37E-02	7	1,623,723

CDYL	0.81	2.18E-04	4.37E-02	35	4,844,693
RGS10	-1.57	2.20E-04	4.37E-02	28	29,708,434
LCK	-1.43	2.22E-04	4.37E-02	2	68,938,292
TRBV28	-2.52	2.22E-04	4.37E-02	16	6,869,037
ABCA4	-2.43	2.36E-04	4.48E-02	6	55,058,361
IL4R	-0.96	2.37E-04	4.48E-02	6	19,253,631
AASS	-2.69	2.37E-04	4.48E-02	14	59,948,143
CXCR3	1.72	2.39E-04	4.48E-02	X	55,882,433
GNGT2	0.73	2.45E-04	4.48E-02	9	25,369,751
PRF1	-2.27	2.48E-04	4.48E-02	4	21,497,537
IGLV1-50	-3.53	2.53E-04	4.48E-02	26	25,571,077
HTRA1	-1.21	2.57E-04	4.48E-02	28	32,131,715
TNFRSF18	-1.72	2.58E-04	4.48E-02	5	56,394,294
MFAP3	0.7	2.61E-04	4.48E-02	4	55,609,790
FLT3	-1.86	2.62E-04	4.48E-02	25	11,594,146
PTPRE	-1.26	2.78E-04	4.66E-02	28	36,746,822
LY49	-3.13	2.79E-04	4.66E-02	27	35,144,651
RUNX2	-1.58	2.80E-04	4.66E-02	12	13,786,142
LAT	-1.95	2.94E-04	4.77E-02	6	18,458,571
OSBP2	-2.8	2.97E-04	4.77E-02	26	23,761,633
IL2RB	-1.59	2.97E-04	4.77E-02	10	27,370,669
