# Supplementary material for: Genome-wide Association Study Identifies Shared Risk Loci Common to Two Malignancies in Golden Retrievers
Source: PLoS Genet. 2015 Feb 2;11(2):e1004922. doi: 10.1371/journal.pgen.1004922 (PMC4333733; doi:10.1371/journal.pgen.1004922)
Supplement: S7 Table — Biological functions predicted by IPA to be altered as a result of the differential gene expression seen in tumors that are high-risk at the 33 Mb locus. (PDF) [file pgen.1004922.s011.pdf]

Supplementary Table 7. Significantly affected biological functions downstream of the observed gene expression changes by the 33 Mb risk haplotype

Functions Annotation	# Mole cules	Predicted Activation State	Activation z-score	B-H corrected p-value	Notes	Molecules
quantity of T lymphocytes	19	Decreased	-2.618	1.46E-08	bias	CCL19,CCL5,CCR6,CD5L,CD8A,CD8B,CSF1,CXCL10,CXCR3,FLT3,IL2RB,LAT,LCK,PRF1, PTPN22,RUNX2,TNFRSF21,TNFRSF4,ZAP70
quantity of mononuclear leukocytes	21	Decreased	-2.897	2.96E-08	bias	CCL19,CCL5,CCR6,CD5L,CD8A,CD8B,CSF1,CXCL10,CXCR3,FLT3,IL2RB,LAT,LCK,PRF1, PROK2,PTPN22,RUNX2,TNFAIP3,TNFRSF21,TNFRSF4,ZAP70
activation of cells	24	Decreased	-3.553	3.72E-08	bias	CCL22,CCL5,CCR6,CD8A,CHGA,CSF1,CXCL10,FLT3,GZMA,GZMB,IL2RB,KLRC4-KLRK1/KLRK1,LAT,LCK,PRF1,PTH1R,PTPN22,PTPRE,RUNX2,TBXA2R,TNFRSF18, TNFRSF21TNFRSF4,ZAP70
activation of T lymphocytes	15	Decreased	-3.070	3.72E-08	bias	CCL22,CCL5,CD8A,CXCL10,GZMA,IL2RB,KLRC4-KLRK1/KLRK1,LAT,LCK,PRF1,PTPN22,TNFRSF18,TNFRSF21,TNFRSF4,ZAP70
quantity of lymphocytes	20	Decreased	-2.727	3.72E-08	bias	CCL19,CCL5,CCR6,CD5L,CD8A,CD8B,CSF1,CXCL10,CXCR3,FLT3,IL2RB,LAT,LCK,PRF1, PTPN22,RUNX2,TNFAIP3,TNFRSF21,TNFRSF4,ZAP70
quantity of leukocytes	23	Decreased	-2.494	3.72E-08		CCL19,CCL22,CCL5,CCR6,CD5L,CD8A,CD8B,CSF1,CXCL10,CXCR3,FLT3,IL2RB,LAT,LCK, PRF1,PROK2,PTPN22,RGS10,RUNX2,TNFAIP3,TNFRSF21,TNFRSF4,ZAP70
quantity of blood cells	24	Decreased	-2.468	3.72E-08		CCL19,CCL22,CCL5,CCR6,CD5L,CD8A,CD8B,CSF1,CXCL10,CXCR3,FLT3,IL2RB,LAT,LCK, PRF1,PROK2,PTH1R,PTPN22,RGS10,RUNX2,TNFAIP3,TNFRSF21,TNFRSF4,ZAP70
activation of mononuclear leukocytes	17	Decreased	-3.313	4.20E-08	bias	CCL22,CCL5,CD8A,CSF1,CXCL10,FLT3,GZMA,IL2RB,KLRC4-KLRK1/KLRK1,LAT,LCK,PRF1,PTPN22,TNFRSF18,TNFRSF21,TNFRSF4,ZAP70
activation of leukocytes	20	Decreased	-3.709	5.79E-08	bias	CCL22,CCL5,CD8A,CHGA,CSF1,CXCL10,FLT3,GZMA,IL2RB,KLRC4-KLRK1/KLRK1, LAT,LCK,PRF1,PTPN22,PTPRE,TBXA2R,TNFRSF18,TNFRSF21,TNFRSF4,ZAP70

cell movement	33	Decreased	-2.067	1.38E-07	bias	CCL19,CCL22,CCL5,CCR6,CD8A,CHGA,CHRM4,COL17A1,CSF1,CXCL10,CXCR3,EOMES, GZMB,HTRA1,IL2RB,KLRC4-KLRK1/KLRK1,LAT,LCK,MAPK11,MARCKSL1,NPTX2,PLEKHG5, PRF1,PROK2,PTH1R,RGS10,ROR2,RUNX2,TNFAIP3,TNFRSF18,TNFRSF21,TNFRSF4,ZAP70
activation of lymphocytes	16	Decreased	-3.163	1.43E-07	bias	CCL22,CCL5,CD8A,CXCL10,FLT3,GZMA,IL2RB,KLRC4-KLRK1/KLRK1,LAT,LCK,PRF1,PTPN22,TNFRSF18,TNFRSF21,TNFRSF4,ZAP70
migration of cells	31	Decreased	-2.366	1.78E-07	bias	CCL19,CCL22,CCL5,CCR6,CD8A,CHGA,CHRM4,COL17A1,CSF1,CXCL10,CXCR3,EOMES, GZMB,IL2RB,KLRC4-KLRK1/KLRK1,LAT,LCK,MAPK11,MARCKSL1,NPTX2,PLEKHG5, PRF1,PROK2,PTH1R,ROR2,RUNX2,TNFAIP3,TNFRSF18,TNFRSF21,TNFRSF4,ZAP70
mobilization of Ca2+	13	Decreased	-2.131	1.85E-06	bias	CCL19,CCL22,CCL5,CCR6,CD8A,CXCL10,CXCR3,KLRC4-KLRK1/KLRK1,LAT,LCK,PROK2,TBXA2R,ZAP70
cell movement of leukemia cell lines	8	Decreased	-2.020	4.30E-06	bias	CCL19,CCL22,CCL5,CSF1,CXCL10,KLRC4-KLRK1/KLRK1,LCK,MAPK11
cell movement of leukocyte cell lines	7	Decreased	-2.410	5.51E-06	bias	CCL19,CCL22,CCL5,CSF1,CXCR3,LCK,ZAP70
quantity of cells	26	Decreased	-2.833	8.18E-06		ABCA4,CCL19,CCL22,CCL5,CCR6,CD5L,CD8A,CD8B,CSF1,CXCL10,CXCR3,FLT3,IL2RB,LAT, LCK,PRF1,PROK2,PTH1R,PTPN22,RGS10,ROR2,RUNX2,TNFAIP3,TNFRSF21,TNFRSF4, ZAP70
stimulation of cells	11	Decreased	-2.340	1.70E-05	bias	CCL22,CCL5,CD8A,CSF1,CXCL10,IL2RB,KLRC4-KLRK1/KLRK1,LCK,RUNX2,TNFAIP3,TNFRSF18
quantity of Ca2+	13	Decreased	-2.777	2.14E-05	bias	CCL19,CCL22,CCL5,CD8A,CHGA,CSF1,CXCL10,CXCR3,LAT,LCK,MARCKSL1,PTH1R,TBXA2R
cell movement of hematopoietic progenitor cells	6	Decreased	-2.169	2.29E-05	bias	CCL19,CCL22,CCL5,CXCL10,CXCR3,ZAP70
quantity of thymocytes	9	Decreased	-2.397	2.48E-05	bias	CCR6,CD5L,CD8A,CD8B,IL2RB,LAT,LCK,RUNX2,ZAP70
quantity of hematopoietic progenitor cells	12	Decreased	-2.613	3.12E-05		CCR6,CD5L,CD8A,CD8B,CSF1,FLT3,IL2RB,LAT,LCK,PTH1R,RUNX2,ZAP70

development of mononuclear leukocytes	13	Decreased	-2.042	6.76E-05	bias	CD8A,CD8B,CSF1,EOMES,FLT3,IL2RB,LAT,LCK,PTPN22,RUNX2,TNFAIP3,TNFRSF4,ZAP70
cytotoxicity of lymphocytes	7	Decreased	-2.367	1.27E-04	bias	CCL5,GZMA,GZMB,KLRC4-KLRK1/KLRK1,LAT,LCK,PRF1
chemotaxis of leukemia cell lines	5	Decreased	-2.191	1.45E-04	bias	CCL19,CCL22,CCL5,CSF1,LCK
metabolism of reactive oxygen species	13	Decreased	-2.950	1.61E-04		CCL5,CSF1,FLT3,GSTA4,GZMA,GZMB,GZMK,LAT,LCK,MAPK11,PRF1,TBXA2R,ZAP70
recruitment of antigen presenting cells	6	Decreased	-2.191	1.66E-04	bias	CCL19,CCL22,CCL5,CD8A,CSF1,CXCL10
cytotoxicity of cells	8	Decreased	-2.242	1.73E-04	bias	CCL5,GZMA,GZMB,KLRC4-KLRK1/KLRK1,LAT,LCK,PRF1,TNFAIP3
polarization of blood cells	6	Decreased	-2.429	1.81E-04	bias	CCL19,CCL22,CCL5,CSF1,CXCL10,LCK
differentiation of cells	26	Decreased	-2.148	1.99E-04		CCL19,CCL5,CD8A,CD8B,CSF1,CXCL10,EOMES,EXTL1,FLT3,GRHL3,GZMB,HTRA1,IL2RB,LAT,LBH,LCK,MAPK11,PTH1R,PTPN22,PTPRE,RGS10,ROR2,RUNX2,TNFAIP3,TNFRSF4,ZAP70
differentiation of hematopoietic progenitor cells	8	Decreased	-2.015	2.25E-04	bias	CD8B,CSF1,EXTL1,FLT3,LAT,LCK,RUNX2,ZAP70
cell viability	19	Decreased	-2.173	3.57E-04	bias	CCL5,CD8A,COL17A1,CSF1,CXCL10,CXCR3,FLT3,IL2RB,LAT,LCK,MAPK11,PROK2,PTH1R,PTPN22,PTPRE,ROR2,TNFAIP3,TNFRSF18,TNFRSF4
synthesis of reactive oxygen species	12	Decreased	-2.801	4.42E-04		CCL5,CSF1,FLT3,GZMA,GZMB,GZMK,LAT,LCK,MAPK11,PRF1,TBXA2R,ZAP70
tyrosine phosphorylation of protein	7	Decreased	-2.445	4.99E-04	bias	CCL5,CD8A,CD8B,CSF1,LCK,PTPN22,ZAP70
length of long bones	4	Decreased	-2.000	7.76E-04	bias	CSF1,PTH1R,ROR2,RUNX2

maturation of cells	10	Decreased	-2.122	8.82E-04	bias	CCL19,CCL5,CD5L,CSF1,EOMES,FLT3,LAT,LCK,PTH1R,RUNX2
polarization of leukocytes	5	Decreased	-2.224	1.40E-03	bias	CCL19,CCL22,CCL5,CSF1,LCK
infection of mammalia	8	Increased	2.019	2.02E-03	bias	CCR6,CD5L,CD8A,CXCL10,CXCR3,GZMA,PADI4,PRF1
production of reactive oxygen species	9	Decreased	-2.953	2.83E-03		CCL5,CSF1,FLT3,GZMA,GZMB,GZMK,LAT,PRF1,ZAP70
activation of myeloid cells	8	Decreased	-2.558	2.97E-03	bias	CCL22,CCL5,CHGA,CSF1,CXCL10,GZMA,PTPRE,TBXA2R
activation of phagocytes	8	Decreased	-2.559	4.07E-03	bias	CCL22,CCL5,CHGA,CSF1,CXCL10,GZMA,PTPRE,TBXA2R
release of Ca2+	6	Decreased	-2.000	5.16E-03	bias	CCL19,CCL5,CHGA,CSF1,LCK,ZAP70
maturation of leukocytes	6	Decreased	-2.404	6.60E-03	bias	CCL19,CCL5,CSF1,EOMES,LAT,LCK
phosphorylation of protein	10	Decreased	-2.445	1.03E-02	bias	CCL5,CD8A,CD8B,CSF1,FLT3,LCK,MAPK11,PTPN22,TNIK,ZAP70
cell movement of tumor cell lines	11	Decreased	-2.292	1.13E-02	bias	CCL19,CCL22,CCL5,CSF1,CXCL10,GZMB,HTRA1,KLRC4-KLRK1/KLRK1,LCK,MAPK11,RUNX2
proliferation of leukocyte cell lines	5	Decreased	-2.067	1.16E-02	bias	CD8A,CSF1,FLT3,IL2RB,LCK
