# Supplementary material for: Genome-wide Association Study Identifies Shared Risk Loci Common to Two Malignancies in Golden Retrievers
Source: PLoS Genet. 2015 Feb 2;11(2):e1004922. doi: 10.1371/journal.pgen.1004922 (PMC4333733; doi:10.1371/journal.pgen.1004922)
Supplement: S8 Table — Canonical pathways estimated by IPA to be affected as a result of the differential gene expression seen in tumors that are high-risk at the 33 Mb locus. (PDF) [file pgen.1004922.s012.pdf]

Supplementary Table 8. Canonical pathways with significant ($p < 0.05$) enrichment of the genes with expression changes by the 33 Mb risk haplotype

Ingenuity Canonical Pathways	B-H p-value	Ratio	Molecules
CTLA4 Signaling in Cytotoxic T Lymphocytes	1.58E-04	0.07	LCK,LAT,ZAP70,CD8A,CD8B,PTPN22
Pathogenesis of Multiple Sclerosis	3.39E-04	0.33	CXCL10,CXCR3,CCL5
T Cell Receptor Signaling	2.24E-03	0.05	LCK,LAT,ZAP70,CD8A,CD8B
IL-17A Signaling in Gastric Cells	4.47E-03	0.12	CXCL10,CCL5,MAPK11
Communication between Innate and Adaptive Immune Cells	0.01	0.04	CXCL10,CCL5,CD8A,CD8B
Granulocyte Adhesion and Diapedesis	0.01	0.03	CXCL10,CCL22,CCL5,MMP25,CCL19
Agranulocyte Adhesion and Diapedesis	0.01	0.03	CXCL10,CCL22,CCL5,MMP25,CCL19
iCOS-iCOSL Signaling in T Helper Cells	0.01	0.04	LCK,LAT,ZAP70,IL2RB
Natural Killer Cell Signaling	0.01	0.03	LCK,KLRC4-KLRK1/KLRK1,LAT,ZAP70
Hematopoiesis from Pluripotent Stem Cells	0.01	0.06	CSF1,CD8A,CD8B
Primary Immunodeficiency Signaling	0.01	0.06	LCK,ZAP70,CD8A
Granzyme B Signaling	0.02	0.13	PRF1,GZMB
IL-15 Signaling	0.03	0.04	LCK,MAPK11,IL2RB
Role of MAPK Signaling in the Pathogenesis of Influenza	0.03	0.04	CXCL10,CCL5,MAPK11
Granzyme A Signaling	0.03	0.10	GZMA,PRF1
VDR/RXR Activation	0.03	0.04	CXCL10,RUNX2,CCL5
Tumoricidal Function of Hepatic Natural Killer Cells	0.03	0.08	PRF1,GZMB
Crosstalk between Dendritic Cells and Natural Killer Cells	0.04	0.03	PRF1,KLRC4-KLRK1/KLRK1,IL2RB
