# Supplementary material for: Genome-wide Association Study Identifies Shared Risk Loci Common to Two Malignancies in Golden Retrievers
Source: PLoS Genet. 2015 Feb 2;11(2):e1004922. doi: 10.1371/journal.pgen.1004922 (PMC4333733; doi:10.1371/journal.pgen.1004922)
Supplement: S9 Table — Upstream regulators suggested by IPA to explain the differential gene expression seen in tumors that are high-risk at the 33 Mb locus. (PDF) [file pgen.1004922.s013.pdf]

Supplementary Table 9. Upstream regulators of the observed gene expression changes by the 33 Mb risk haplotype

Upstream Regulator	Molecule Type	Predicted Activation State	Activation z-score	p-value of overlap (Fisher's exact)	Notes	Target molecules in dataset
IL2	cytokine	Inhibited	-2.967	5.62E-14	bias	CCL5,CCR6,CD8A,CSF1,CXCL10,CXCR3,EOMES,GZMA,GZMB,IL2RB,KLRC4-KLRK1/KLRK1,LCK,MMP25,PRF1,PROK2,TNFRSF18,TNFRSF21,TNFRSF4
CD3	complex	Activated	2.022	3.34E-13	bias	CCL22,CCL5,CCR6,CD8A,CD8B,CXCL10,CXCR3,EOMES,GZMA,GZMB,GZMK,IL2RB,LCK,PRF1,PTPN22,STOM,TNFAIP3,TNFRSF18,TNFRSF4
TCR	complex	Inhibited	-2.833	6.31E-13	bias	CCL22,CCL5,CCR6,CD8B,CXCL10,CXCR3,GZMA,GZMB,LAT,LCK,PRF1,TNFRSF18,TNFRSF4,ZAP70
ZBTB7B	transcription regulator	Activated	2.207	1.13E-09		CCR6,CD8A,CD8B,EOMES,IL2RB,PRF1
IL15	cytokine	Inhibited	-2.632	2.96E-09	bias	CCL19,CCL5,CD8A,CD8B,CSF1,CXCL10,GZMB,GZMK,IL2RB,KLRC4-KLRK1/KLRK1,LCK,PRF1
CD40LG	cytokine	Inhibited	-2.209	8.31E-08	bias	CCL19,CCL22,CCL5,CD8A,CSF1,CXCL10,CXCR3,GZMB,MARCKSL1,TNFAIP3,ZAP70
NFKB1	transcription regulator	Inhibited	-2.2	1.03E-05	bias	CCL19,CCL22,CCL5,CSF1,CXCL10,TNFAIP3,TNFRSF4
miR-1237-3p (and other miRNAs w/seed CCUUCUG)	mature microRNA	Activated	3.09	4.52E-05	bias	ABCA4,CCL19,CNNM1,CORO6,EOMES,IL2RB,OBSL1,PROK2,RUNX2,TNIK
IL18	cytokine	Inhibited	-2.219	9.82E-05	bias	CCL5,CSF1,CXCL10,KLRC4-KLRK1/KLRK1,PRF1
CBFB	transcription regulator	Activated	2	9.85E-05		CCR6,IL2RB,TNFRSF18,TNFRSF4
miR-382-5p (miRNAs w/seed AAGUUGU)	mature microRNA	Activated	2.177	1.52E-04	bias	AFF2,GALNT13,HS3ST3B1,KIAA1598,KRT24,LAD1,MARCKSL1,SMOC1
TNF	cytokine	Inhibited	-2.213	1.97E-04	bias	CCL19,CCL22,CCL5,CCR6,CSF1,CXCL10,CXCR3,LAD1,MARCKSL1,PROK2,RUNX2,TNFAIP3,TNFRSF18,TNFRSF21,TNFRSF4

GLI1	transcription regulator	Inhibited	-2.219	4.39E-04	bias	MAPK11,NPDC1,PTH1R,RGS10,RUNX2
miR-4648 (miRNAs w/seed GUGGGAC)	mature microRNA	Activated	2.17	5.96E-04	bias	AFF2,CCL22,KLRC4-KLRK1/KLRK1,MMP25,NKG7,OBSL1,PLEKHG5,PTPRE,SCN2A
RELA	transcription regulator		-2	1.16E-03	bias	CCL19,CCL22,CCL5,CXCL10,TNFAIP3,TNFRSF4
IKBKB	kinase	Inhibited	-2.194	1.52E-03	bias	CCL19,CCL5,CSF1,CXCL10,TNFRSF4
miR-486-3p (and other miRNAs w/seed GGGGCAG)	mature microRNA	Activated	2.814	2.03E-03	bias	CCSER1,CD5L,CORO6,CSF1,CXCR3,GALNT13,IL2RB,KLRC4-KLRK1/KLRK1,MARCKSL1,NPTX2,OSBP2,PLEKHG5,SMOC1,TMEM178A,TMPRSS13,TNFRSF4
miR-3151-5p (miRNAs w/seed GUGGGGC)	mature microRNA	Activated	2.408	2.41E-03	bias	CCL22,CCSER1,CHGA,CNNM1,CXCR3,FLT3,HTRA1,IL2RB,NPTX2,OSBP2,PIP5K1B,SMOC1,ZAP70
miR-3678-3p (miRNAs w/seed UGCAGAG)	mature microRNA	Activated	2.759	2.70E-03	bias	CCL22,CD5L,CORO6,CXCL10,IL2RB,LCK,PTPRE,TNIK
miR-1908-5p (and other miRNAs w/seed GGCGGGG)	mature microRNA	Activated	3.248	2.79E-03	bias	CCL22,CD5L,CD8A,CORO6,CSF1,EXTL1,MMP25,NPTX2,PLEKHG5,ROR2,TNFRSF4
miR-519a-3p (and other miRNAs w/seed AAGUGCA)	mature microRNA	Activated	2.8	2.87E-03	bias	CSF1,GALNT13,GZMK,KIAA1598,SLC25A48,SMOC1,STOM,TNFRSF21
miR-3978 (miRNAs w/seed UGGAAAG)	mature microRNA	Activated	2.425	4.43E-03	bias	LBH,OSBP2,PROK2,RUNX2,STOM,TNFAIP3
miR-125b-5p (and other miRNAs w/seed CCCUGAG)	mature microRNA	Activated	2.266	5.91E-03	bias	CD5L,CD8A,CNNM1,HIST1H4L,IL2RB,KIAA1598,LBH,MMP25,PRF1,PTH1R,TMPRSS13,TNFAIP3
miR-4300 (and other miRNAs w/seed GGGAGCU)	mature microRNA	Activated	3.162	7.21E-03	bias	CCL22,CD8B,EXTL1,GALNT13,IL2RB,LAD1,NPTX2,OSBP2,TBXA2R,TNFRSF21
miR-4802-3p (and other miRNAs w/seed ACAUGGA)	mature microRNA	Activated	2.619	8.66E-03	bias	CCSER1,CD5L,KIAA1598,MMP25,PTPRE,ROR2,STOM

miR-1225-3p (miRNAs w/seed GAGCCCC)	mature microRNA	Activated	3.092	9.40E-03	bias	CCL22,CD5L,CD8B,CORO6,IL2RB,LAT,MMP25,NPTX2,OSBP2,ROR2
miR-1237-5p (and other miRNAs w/seed GGGGGCG)	mature microRNA	Activated	3.405	1.27E-02	bias	CD5L,CD8B,CHGA,CORO6,EXTL1,IL2RB,KIAA1598,MMP25,NPTX2,OBSL1,OSBP2,PTPRE
miR-423-5p (and other miRNAs w/seed GAGGGGC)	mature microRNA	Activated	2.892	1.60E-02	bias	ACTL7A,CCL22,CCL5,CD8A,CSF1,DLGAP3,LAD1,NKG7,NPTX2,OBSL1,OSBP2,TBXA2R
miR-935 (miRNAs w/seed CAGUUAC)	mature microRNA	Activated	2.219	1.62E-02	bias	C17orf104,EOMES,GALNT13,IL2RB,PROK2
miR-3090-3p (and other miRNAs w/seed CCCAGGU)	mature microRNA	Activated	2.401	1.88E-02	bias	C17orf104,CCL19,CCL22,CD8A,CORO6,LAT,MMP25,OSBP2,STOM,TMPRSS13
miR-1915-3p (and other miRNAs w/seed CCCAGGG)	mature microRNA	Activated	2.761	2.03E-02	bias	CCL22,CCR6,CD8A,IL2RB,LAD1,LAT,LCK,MMP25,NPDC1,NPTX2,OBSL1,SMOC1
miR-1247-5p (miRNAs w/seed CCCGUCC)	mature microRNA	Activated	2.2	2.07E-02	bias	CD8A,LBH,NPTX2,TNFRSF18,TNFRSF4
miR-3656 (and other miRNAs w/seed GCGGGUG)	mature microRNA	Activated	2.2	2.25E-02	bias	CCL5,CD8A,CSF1,EXTL1,MMP25
miR-663b (miRNAs w/seed GUGGCCC)	mature microRNA	Activated	2.373	2.43E-02	bias	AASS,CNNM1,GALNT13,HIST1H4L,KLRC4-KLRK1/KLRK1,MMP25,PLEKHG5,RUNX2,TNFRSF21
miR-1285-3p (and other miRNAs w/seed CUGGGCA)	mature microRNA	Activated	3.251	2.44E-02	bias	CCL22,CD8A,CNNM1,LAD1,MAPK11,MARCKSL1,NPTX2,PLEKHG5,RUNX2,STOM,TNFRSF21
miR-204-5p (and other miRNAs w/seed UCCCUUU)	mature microRNA	Activated	2.124	2.46E-02	bias	CCSER1,CD8B,COL17A1,FLT3,ROR2,RUNX2,SCN2A,SMOC1,TNFAIP3
miR-4260 (and other miRNAs w/seed UUGGGGC)	mature microRNA	Activated	2.582	2.46E-02	bias	AFF2,CCL22,CD5L,GALNT13,NPTX2,OSBP2,SMOC1

miR-4536-5p (miRNAs w/seed GUGGUAG)	mature microRNA	Activated	2.183	2.73E-02	bias	CCL22,CCL5,KIAA1598,STOM,TNIK
miR-4537 (miRNAs w/seed GAGCCGA)	mature microRNA	Activated	2.194	2.88E-02	bias	AASS,CSF1,LAD1,PLEKHG5,STOM
miR-4751 (miRNAs w/seed GAGGACC)	mature microRNA	Activated	2.401	2.99E-02	bias	EXTL1,GALNT13,IL2RB,LAT,MAPK11,RUNX2
miR-711 (miRNAs w/seed GGACCCA)	mature microRNA	Activated	2.587	3.26E-02	bias	AFF2,CD8B,CNNM1,GSTA4,GZMK,KLRC4-KLRK1/KLRK1,TBXA2R
miR-331-3p (miRNAs w/seed CCCCUGG)	mature microRNA	Activated	2.155	4.02E-02	bias	CCSER1,CNNM1,EOMES,GALNT13,KLRC4-KLRK1/KLRK1,LAD1,LBH,MAPK11,TMPRSS13
miR-2392 (miRNAs w/seed AGGAUGG)	mature microRNA	Activated	2.186	4.13E-02	bias	CHGA,CXCL10,KLRC4-KLRK1/KLRK1,LAT,PLEKHG5
miR-3614-3p (miRNAs w/seed AGCCUUC)	mature microRNA	Activated	2.169	4.23E-02	bias	CNNM1,GALNT13,RUNX2,TNFAIP3,TNIK
miR-3594-5p (and other miRNAs w/seed CCAGGGC)	mature microRNA	Activated	3.247	4.25E-02	bias	AFF2,CCL22,CD8A,CD8B,CHGA,CSF1,MAPK11,MARCKSL1,PLEKHG5,SMOC1,TBXA2R
miR-4650-3p (miRNAs w/seed GGUAGAA)	mature microRNA	Activated	2.236	4.29E-02	bias	AASS,CCL5,FLT3,GALNT13,GZMK
miR-3657 (and other miRNAs w/seed GUGUCCC)	mature microRNA	Activated	2.383	4.44E-02	bias	AFF2,CD8B,CNNM1,COL17A1,CSF1,EXTL1
miR-4324 (miRNAs w/seed CCUGAGA)	mature microRNA	Activated	2.425	4.60E-02	bias	C17orf104,CCL22,CD5L,LAD1,RGS10,SMOC1
miR-4640-5p (and other miRNAs w/seed GGGCCAG)	mature microRNA	Activated	3.286	4.64E-02	bias	CCL22,CD5L,CNNM1,CSF1,EXTL1,MARCKSL1,OBSL1,OSBP2,PRF1,RUNX2,SMOC1
miR-2355-3p (miRNAs w/seed UUGUCCU)	mature microRNA	Activated	2.183	4.92E-02	bias	AASS,KLRC4-KLRK1/KLRK1,MMP25,TMEM178A,TNIK

miR-1273h-5p (and other miRNAs w/seed UGGGAGG)	mature microRNA	Activated	3.069	4.95E-02	bias	AASS,AFF2,CCL22,CNNM1,CXCL10,GRHL3,MARCKSL1,TBXA2R,TMEM178A,TNIK
